# Solar‐Powered Hierarchical Microenvironments with Authigenic Multi‐Field Synergies for Simultaneous Extraction of Freshwater and Cesium

**DOI:** 10.1002/advs.202505997

**Published:** 2025-05-11

**Authors:** Liuyan Zhu, Lin Zhu, Ting Shi, Ke Zhao, Wenwen He, Lu Han, Zeying Jin, Jialin Kang, Shanfu Sun, Ningning Cao, Zhen Yu

**Affiliations:** ^1^ Hebei Key Laboratory of Active Components and Functions in Natural Products Hebei Normal University of Science & Technology Qinhuangdao 066004 China; ^2^ Air Defense and Antimissile School Air Force Engineering University Xi'an 710100 China; ^3^ Instrumental Analysis & Research Center Sun Yat‐Sen University Shenzhen 518107 China; ^4^ School of Chemistry and Chemical Engineering Beijing Institute of Technology Beijing 102488 China; ^5^ School of Aerospace Science and Technology Xidian University Xi'an 710126 China; ^6^ College of Engineering and Applied Sciences Nanjing University Nanjing 210093 China; ^7^ Department of Mechanical Engineering City University of Hong Kong Hong Kong 999077 China

**Keywords:** adsorption, cesium extraction, freshwater production, radioactive substance, solar‐powered interfacial evaporation

## Abstract

The escalating global resource and environmental crisis has intensified the urgent demand for efficiently extracting freshwater and critical metals from contaminated water sources, particularly nuclear‐contaminated seawater. Herein, a biomass‐based solar‐powered hierarchical self‐extraction micro‐network (BSSM) is proposed and presented, an innovative system that seamlessly integrates solar‐driven freshwater generation with highly selective cesium ion (Cs^+^) extraction. A bamboo‐derived biochar with surface‐loaded cobalt oxide nanoparticles (CoO NPs) is prepared and utilized to comprehensively validate the BSSM's efficacy. Leveraging hierarchically engineered microenvironments with authigenic multi‐field synergies, the designed BSSM achieves a high freshwater production rate of 3.3 kg m^−2^ day^−1^ and an impressive Cs^+^ extraction capacity of 34.58 mg g^−1^. In conclusion, this study establishes a transformative paradigm for resource recovery, providing a scalable and eco‐friendly panacea to address pressing global issues, including water scarcity, nuclear pollution, and resource security.

## Graphical abstract

A biomass‐based solar‐powered hierarchical self‐extraction micro‐network (BSSM) is first proposed to achieve freshwater production and Cs^+^ extraction. This study establishes a transformative paradigm for resource recovery, providing a scalable and eco‐friendly panacea to address pressing global issues, including water scarcity, nuclear pollution, and resource security.

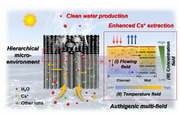

## 1 Introduction

**Figure 1:**
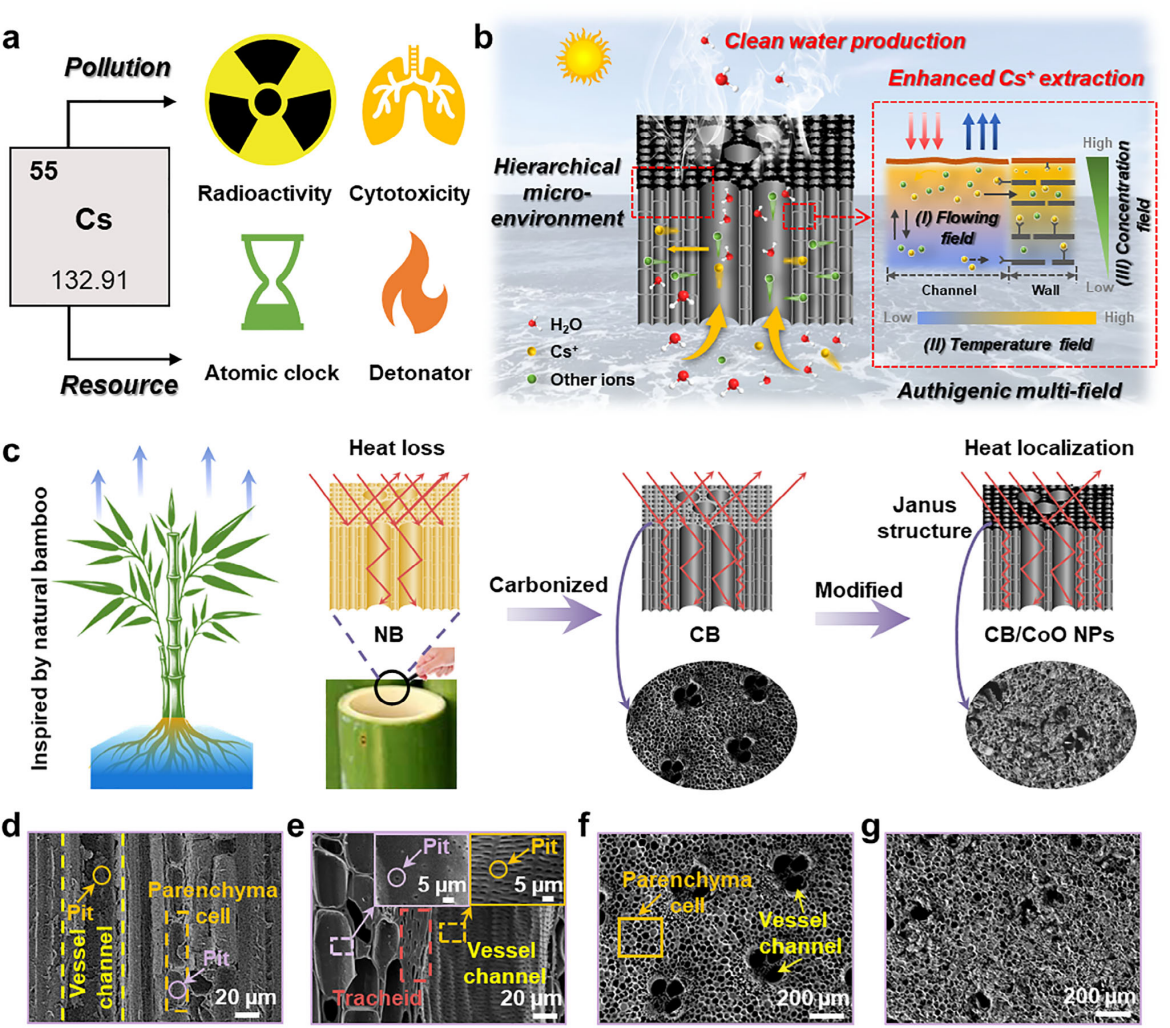
a) Overview of Cs element: environmental impacts, health hazards, and resource utilization. b) The working principle of the designed BSSM. c) Schematic showing the BSSM assembly process inspired by natural bamboo. d,e) SEM images of the longitudinal cross‐sections of NB and CB demonstrate CB's hierarchical structure retention. f,g) SEM images of CB and CB/CoO NPs‐based BSSM.

The ocean, functioning as the most critical water resource reservoir and the pillar of the ecosystem on Earth, is now confronting significant and escalating challenges due to seawater pollution, particularly from events such as nuclear leakage.^[^ ^1^, ^2^, ^3^ ^]^ Nuclear‐contaminated seawater harbors a variety of radioactive substances, among which cesium, particularly cesium‐137 (^137^Cs),^[^ ^4^, ^5^, ^6^, ^7^ ^]^ stands out as a quintessential example (**Figure** **1**a). With a long half‐life of up to 30 years, ^137^Cs emits β and γ rays, which can directly damage human tissues, increasing the risk of cancer and genetic mutations. Moreover, its high solubility and mobility enable bioaccumulation through the food chain, posing long‐term threats to human health.^[^ ^5^ ^]^ The environmental persistence and global dispersion of ^137^Cs further exacerbate its ecological impact, making its containment and recovery a critical priority. Despite these challenges, cesium also holds significant resource potential. The unique hyperfine energy‐level structure of cesium atoms makes it an indispensable material for high‐precision atomic clocks, vital for satellite navigation, communication, and astronomical observation.^[^ ^6^ ^]^ In aerospace, the cesium‐ion propulsion technology, renowned for its high specific impulse and propulsion efficiency compared to traditional chemical propellants, thereby offers a revolutionary solution for the deep‐space exploration and orbit maintenance.^[^ ^8^, ^9^ ^]^ Beyond the above applications, cesium also plays a key role in medical, energy, and scientific research fields.^[^ ^10^ ^]^ As such, the simultaneous extraction of freshwater and cesium from nuclear‐contaminated seawater represents not only an urgent response to water scarcity and nuclear pollution mitigation but also a pivotal pathway toward achieving resource recycling and sustainable development.

Currently, the primary methods for extracting freshwater from seawater include distillation, reverse osmosis, and electrodialysis.^[^ ^11^, ^12^, ^13^ ^]^ While distillation is a well‐established technology, it is hampered by high energy demands and substantial capital costs. Reverse osmosis, showing relatively low energy consumption, is limited by the issues of membrane fouling, low flow rates, as well as suboptimal processing efficiency. Electrodialysis, in sharp contrast, is restricted to low‐salinity seawater and is further challenged by high initial investment, membrane fouling, incomplete technological maturation, and stringent water quality requirements.^[^ ^14^ ^]^ For cesium extraction from seawater, the predominant techniques are chemical precipitation, ion exchange, and solvent extraction. Among them, chemical precipitation, while operationally simple and cost‐effective, suffers from poor selectivity and a tendency for co‐precipitation with other ions.^[^ ^6^ ^]^ Ion exchange offers high selectivity but is constrained by the susceptibility of ion exchange resins to fouling and the complexity of the regeneration process.^[^ ^15^ ^]^ Despite its high efficiency, solvent extraction involves intricate operational procedures and raises environmental concerns due to the usage of organic solvents.^[^ ^11^ ^]^ As summarized above and in Table S1 (Supporting Information), existing technologies for seawater desalination or cesium extraction remain constrained by inherent limitations. To date, no single technology or integrated system has been developed to achieve the simultaneous, sustainable, and environmentally benign co‐extraction of freshwater and cesium—particularly from nuclear‐contaminated seawater—highlighting a critical technological gap.

Solar‐driven interfacial evaporation technology represents a sustainable and eco‐friendly solution to generate freshwater.^[^ ^16^, ^17^, ^18^, ^19^, ^20^ ^]^ Further, through the integration design of photothermal materials with advanced selective adsorbents, the evaporator can facilitate the simultaneous recovery of freshwater and various strategic metals, such as lithium and uranium.^[^ ^4^, ^21^, ^22^ ^]^ However, related research on the co‐extraction of freshwater and cesium resources remains scarce, especially those utilizing biomass‐based evaporators. As such, further in‐depth studies are essential to fill such technical gaps and unlock the full potential of this promising technology.

Here, we propose and present a biomass‐based solar‐powered hierarchical self‐extraction micro‐network (BSSM) (Figure 1b), designed to offer a brand‐new solution for simultaneous extraction of freshwater and cesium resources from nuclear‐contaminated seawater. The BSSM ingeniously leverages the unique physiological architecture of natural bamboo (NB) and its intrinsic water and nutrient transport mechanisms, while surface functionalization with cobalt oxide nanoparticles (CoO NPs) induces a Janus structure. Such a rational design enables the synergistic interaction of concentration, temperature, and flow fields within the hierarchical architecture of BSSM, suppressing salt accumulation and meanwhile enhancing Cs^+^ extraction. As we know, it is the first biomass‐based evaporator that enables the simultaneous extraction of freshwater and cesium ions (Cs^+^) (Table S1, Supporting Information). In summary, the designed BSSM presents a high‐efficiency strategy to tackle global water scarcity and reduce risks associated with radioactive contamination, offering far‐reaching implications for environmental sustainability and resource recovery.

## 2 Results and Discussion

### 2.1 Fabrication of the BSSM

The designed BSSM suitably takes advantage of the water or nutrient‐management mechanisms of bamboo (Figure 1c), integrating root‐mediated substance absorption, vessel‐facilitated substance transport, and transpiration‐driven vapor release. Figure 1d illustrates the sophisticated xylem architecture of the natural bamboo (NB), primarily composed of vessels, tracheids (collectively called vascular bundles), and parenchyma cells. The vessel channels function as essential pathways for the upward transport of water and inorganic salts, whereas the parenchyma cells, which contain starch granules that seal cellular pores, are interconnected by pits with diameters of 1–2 µm. This hierarchical microstructure, characterized by its unique longitudinal channels and transverse interconnected networks, facilitates efficient longitudinal transport and transverse diffusion of water and nutrients.

Carbonized bamboo (CB) obtained via carbonization treatment retains the core microstructural features of the original NB, while its fine structure is significantly optimized during the pyrolysis process. The cell walls of the as‐prepared CB become thinner and the surface smoother, while the pit diameters on the inner walls of the vessels increase to 2–3 µm, more prominent compared to the 1–2 µm within the original NB (Figure 1e). Simultaneously, the carbonization process can effectively remove the organic impurities, such as lignin and hemicellulose in the vessels and parenchyma cells, and clear blockages in the pit membranes, thus forming a 3D interconnected hierarchical pore structure. Such structural features facilitate rapid migration and diffusion of salt ions within CB during solar‐powered seawater evaporation. Further research reveals that the vessel system in CB exhibits excellent capillary water‐transport performance (Figure S1, Supporting Information). The water molecules are continuously transported upward from the bottom water body through capillary action, and the equilibrium height (*h*) in the steady state can be calculated according to Jurin's law^[^ ^23^ ^]^:

(1) $$h\;=\frac{2\;\gamma \;\cos\theta }{\rho \;g\;r}$$

where *γ* represents the surface tension of water; *θ* is the contact angle; *r* denotes the vessel's radius; *ρ* is the density of water; and *g* is the gravitational acceleration. Through testing and analysis of the different vessels (Figure S2, Supporting Information), the equilibrium height (*h*) was calculated to be 277.79 cm (Table S2, Supporting Information), significantly higher than the average height of the CB sample (1.0–2.0 cm). This result confirms the high efficiency and reliability of CB vessels in water transport processes.

To further enhance the light absorption and salt resistance of the evaporator, a hydrophobic CoO nanoparticles (CoO NPs) layer was thus stably deposited onto the surface of CB through chemical vapor deposition (CVD), resulting in a CB/CoO NPs composite material with a unique Janus‐structured characteristic. Microstructural characterizations (Figure 1f,g; Figures S3 and S4, Supporting Information) indicate that the vessel channels of CB are orderly arranged, exhibiting a complex hierarchical porous network. After CVD modification, CoO NPs are uniformly distributed on the CB surface at the nanoscale without disrupting the original directional water‐transport channels. Such design is anticipated not only to improve the BSSM's light absorption capacity significantly but also to enhance its long‐term anti‐salt property, thus enabling sustained and stable evaporation performance (detailed analysis will be discussed later).

### 2.2 Characterizations of the BSSM

**Figure 2:**
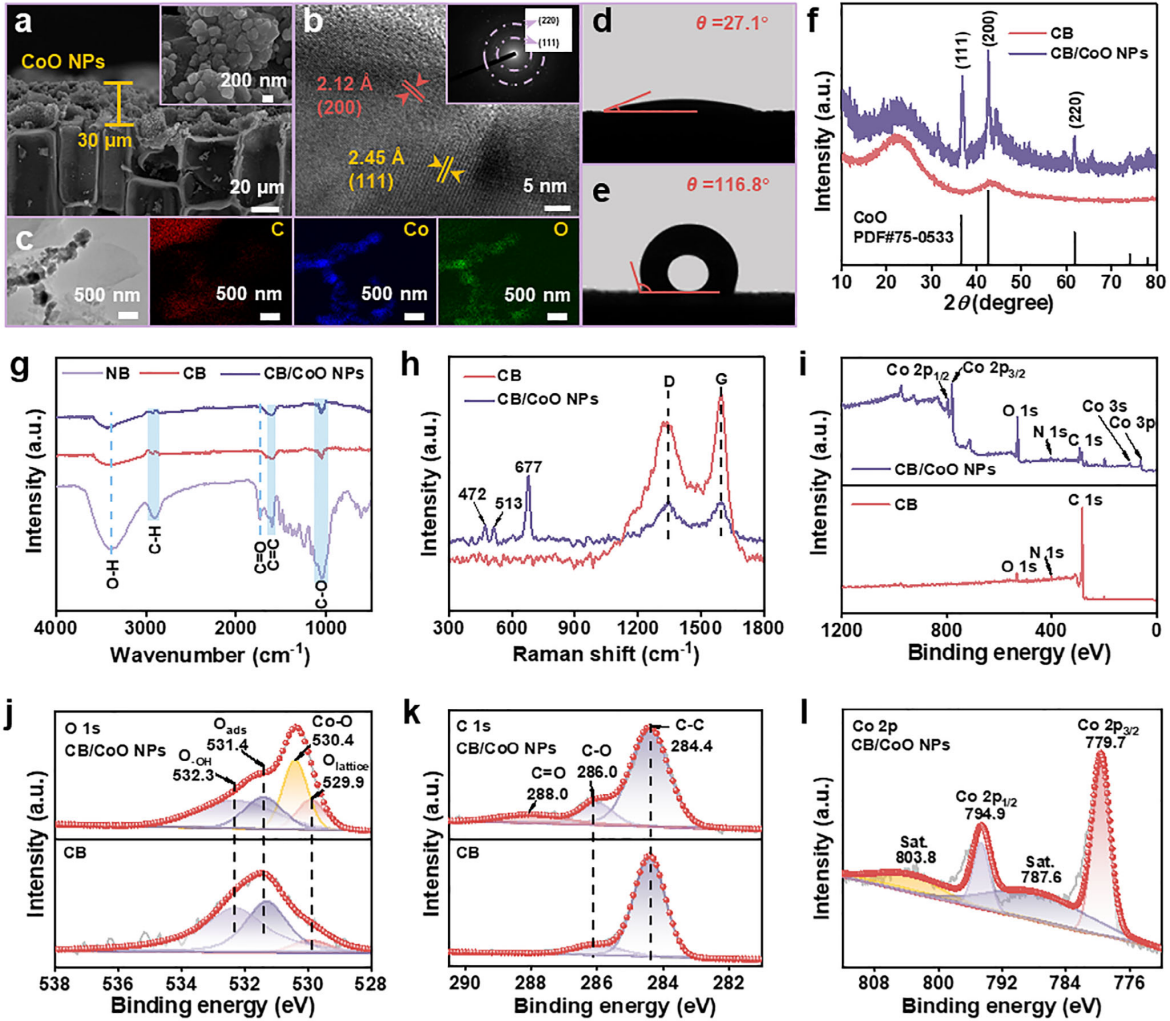
a) SEM image showcasing the longitudinal section of CB/CoO NPs‐based BSSM (inset: high‐magnification view of CoO NPs). b) HR‐TEM image of the CB/CoO NPs‐based BSSM (inset: SAED pattern). c) TEM image and corresponding elemental mappings of CB/CoO NPs. Contact angle measurements for d) the pristine CB and e) the CB/CoO NPs‐based BSSM. f) XRD patterns of CB and the CB/CoO NPs‐based BSSM. g) FT‐IR spectra of NB, CB, and the CB/CoO NPs‐based BSSM. h) Raman spectra of CB and the CB/CoO NPs‐based BSSM. i) XPS survey spectra of CB and the CB/CoO NPs‐based BSSM. j,k) High‐resolution O1s and C1s XPS spectra with peak fitting for CB and the CB/CoO NPs‐based BSSM. l) High‐resolution Co 2p XPS spectrum with peak fitting for the CB/CoO NPs‐based BSSM.

Scanning electron microscopy (SEM) analysis (**Figure** **2**a; Figure S4, Supporting Information) revealed that CoO NPs formed a uniform and dense photothermal conversion layer upon the CB surface, with a thickness of ≈30 µm. The CoO NPs featured a uniform size distribution, with an average diameter of 157.56 ± 59.86 nm (Figure S5, Supporting Information). This nanoscale dimension is conducive to enhancing the surface plasmon resonance effect, thus improving light absorption efficiency. High‐resolution transmission electron microscopy (HR‐TEM) analysis (Figure 2b) unveiled the crystal structure characteristics of CoO NPs, with lattice fringe widths of 2.12 and 2.45 Å, corresponding to the (200) and (111) crystal planes of CoO, respectively. The selected‐area electron diffraction (SAED) pattern (inset in Figure 2b) displayed additional diffraction rings alongside the primary diffraction spots, indicating the presence of abundant grain boundaries and structural defects among CoO NPs. Such features are beneficial for enhancing the separation efficiency of photogenerated carriers. Transmission electron microscopy (TEM) elemental mapping (Figure 2c) also confirmed the successful deposition of CoO NPs onto the CB surface. Additionally, the contact‐angle test results (Figure 2d,e) demonstrated that introducing CoO NPs can significantly alter the surface wettability of BSSM. The surface contact angle of the pristine CB is 27.1°, showing typical hydrophilicity, while that of the CB/CoO NPs‐based BSSM reaches 116.8°, transforming it into a hydrophobic surface. Such a significant wettability change is primarily attributed to the synergistic effects of reduced surface energy and nanoscale roughness of CoO NPs, which is beneficial for inhibiting salt crystallization and thus improving stability.^[^ ^24^, ^25^ ^]^

The crystalline structure of the different samples was characterized using X‐ray diffraction (XRD). As shown in Figure S6 (Supporting Information), the distinct characteristic diffraction peaks in the XRD pattern of NB appeared at 17.5°, 22.4°, and 34.8°, corresponding to the (110), (200), and (004) crystal planes of cellulose, respectively.^[^ ^26^ ^]^ The above result well confirmed the presence of cellulose within NB. However, due to the relatively large bandgap of cellulose (3.5–4.5 eV), its absorption capacity for visible and near‐infrared light is limited. After the carbonization treatment, the characteristic peaks of cellulose disappeared in the resulting CB, and new diffraction peaks emerged at 22.5° and 43.6° (Figure 2f). The above peaks correspond to the (002) and (100) planes of graphitic amorphous carbon,^[^ ^27^ ^]^ indicating the successful structural transformation of cellulose into graphitic carbon during the carbonization process. This transformation would contribute to a significant enhancement in the light absorption capacity of the material. Further analysis of the XRD pattern of the CB/CoO NPs composite system revealed sharp diffraction peaks at 36.9°, 42.7°, and 61.8°, corresponding to the (111), (200), and (220) planes of CoO,^[^ ^28^, ^29^, ^30^ ^]^ respectively, confirming the successful loading of CoO NPs layer onto the CB surface.

FT‐IR spectra (Figure 2g) indicated that after carbonization, the characteristic vibrational peaks of cellulose in NB, including the O‐H stretching at 3394 cm^−1^, C‐H stretching at 2927 cm^−1^, C = O stretching at 1726 cm^−1^, and C‐O stretching at 1053 cm^−1^, are significantly attenuated. Such changes reveal that the functional groups like hydroxyl, alkyl, and carbonyl groups of cellulose undergo pyrolysis reactions during carbonization. Concurrently, the intensity of the absorption peak ≈1660–1650 cm^−1^ increases markedly, attributed to the aromatic C = C skeletal vibrations, clearly suggesting the formation of more conjugated structures.^[^ ^31^, ^32^, ^33^ ^]^ Raman spectroscopy further elucidated the material's structural changes before and after loading the CoO NPs. As shown in Figure 2h, the peak intensities of D‐band (disordered carbon) and G‐band (graphitic carbon) in CB/CoO NPs are significantly reduced, and characteristic peaks of CoO appear at 472 , 513 , and 677 cm^−1^, consistent with previous findings reported by Ravindra,^[^ ^34^ ^]^ confirming the successful incorporation of CoO NPs onto the CB surface. Notably, compared to the pristine CB, the I_D_/I_G_ ratio increases, indicating that introducing CoO NPs promotes the formation of additional carbon defects.^[^ ^35^, ^36^ ^]^

To further investigate the surface chemical states and elemental composition of CB and CB/CoO NPs‐based BSSM, X‐ray photoelectron spectroscopy (XPS) analysis was conducted. As shown in Figure 2i, compared to the pristine CB, the intensity of the C1s peak in CB/CoO NPs decreases, while the O1s peak intensity significantly increases. This notable change is attributed to the successful loading of CoO NPs on the CB surface. In addition to the C1s and O1s characteristic peaks, CB/CoO NPs also exhibit distinct Co characteristic peaks.^[^ ^29^ ^]^ The high‐resolution O1s spectrum (Figure 2j) can be deconvoluted into four characteristic peaks after Gaussian fitting: lattice oxygen (O_lattice_) at 529.9 eV, Co‐O bonds at 530.4 eV, adsorbed oxygen (O_ads_) at 531.4 eV, and surface hydroxyl groups (‐OH) at 532.3 eV.^[^ ^37^ ^]^ Notably, the presence of O_ads_ indicates a significant number of oxygen vacancies in the material, which indeed, under illumination, can facilitate the capture and release of photoelectrons, triggering local electron excitation and relaxation, thereby generating a pronounced localized thermal effect. In the high‐resolution C1s spectrum (Figure 2k), the C‐C signal peak at 284.4 eV broadens, while the intensities of the C‐O and C = O peaks increase. This change is ascribed to the structural reconstruction of the carbon matrix (with an increase in carbon defects) induced by introducing CoO NPs and forming stable C‐O and C = O bonds between CB and CoO NPs, consistent with the Raman spectroscopy analysis. Additionally, the high‐resolution Co 2p spectrum (Figure 2l) can be deconvoluted into four characteristic peaks: 779.7 eV (Co 2p3/2), 794.9 eV (Co 2p1/2), and their corresponding satellite peaks (787.6  and 803.8 eV).^[^ ^38^, ^39^ ^]^ These results further confirm the successful incorporation of CoO NPs onto the CB surface, consistent with the XRD and Raman spectroscopy analyses.

The aforementioned characterization results conclusively demonstrate that hydrophobic CoO NPs have been uniformly and firmly anchored onto the CB surface through chemical bonding, thus constructing a unique Janus bilayer structure. This innovative structural design exhibits significant advantages in multiple aspects: 1) In terms of photothermal conversion, this structure can efficiently convert solar energy into thermal energy and precisely localize the heat at the evaporation interface. This precise heat‐concentration mechanism can significantly improve the utilization efficiency of solar‐thermal energy, ensuring that solar energy is converted and utilized with maximum efficiency, thereby providing a strong impetus for the photothermal conversion process.^[^ ^40^, ^41^ ^]^ 2) Regarding water evaporation, this structural design effectively shortens the residence time of water at the evaporation interface. Optimizing the kinetic process of water molecules increases the saturated vapor pressure at the evaporation interface, markedly accelerating the phase transition of liquid water to vapor, thus achieving rapid water evaporation and improving the efficiency of the entire evaporation process.^[^ ^42^, ^43^, ^44^ ^]^ 3) In terms of anti‐salt and anti‐clogging performance, the hydrophobic surface plays a crucial role.^[^ ^45^ ^]^ It can effectively prevent the accumulation of salts and other impurities from seawater at the evaporation interface, fundamentally avoiding the occurrence of salt crystallization and surface clogging issues.^[^ ^24^, ^25^, ^46^ ^]^ This not only ensures the evaporation interface's cleanliness and unobstructed nature but maintains the BSSM's stability and functionality, thereby guaranteeing long‐term performance stability, as verified in the subsequent part.

### 2.3 Solar‐Powered Water Evaporation Performance

**Figure 3:**
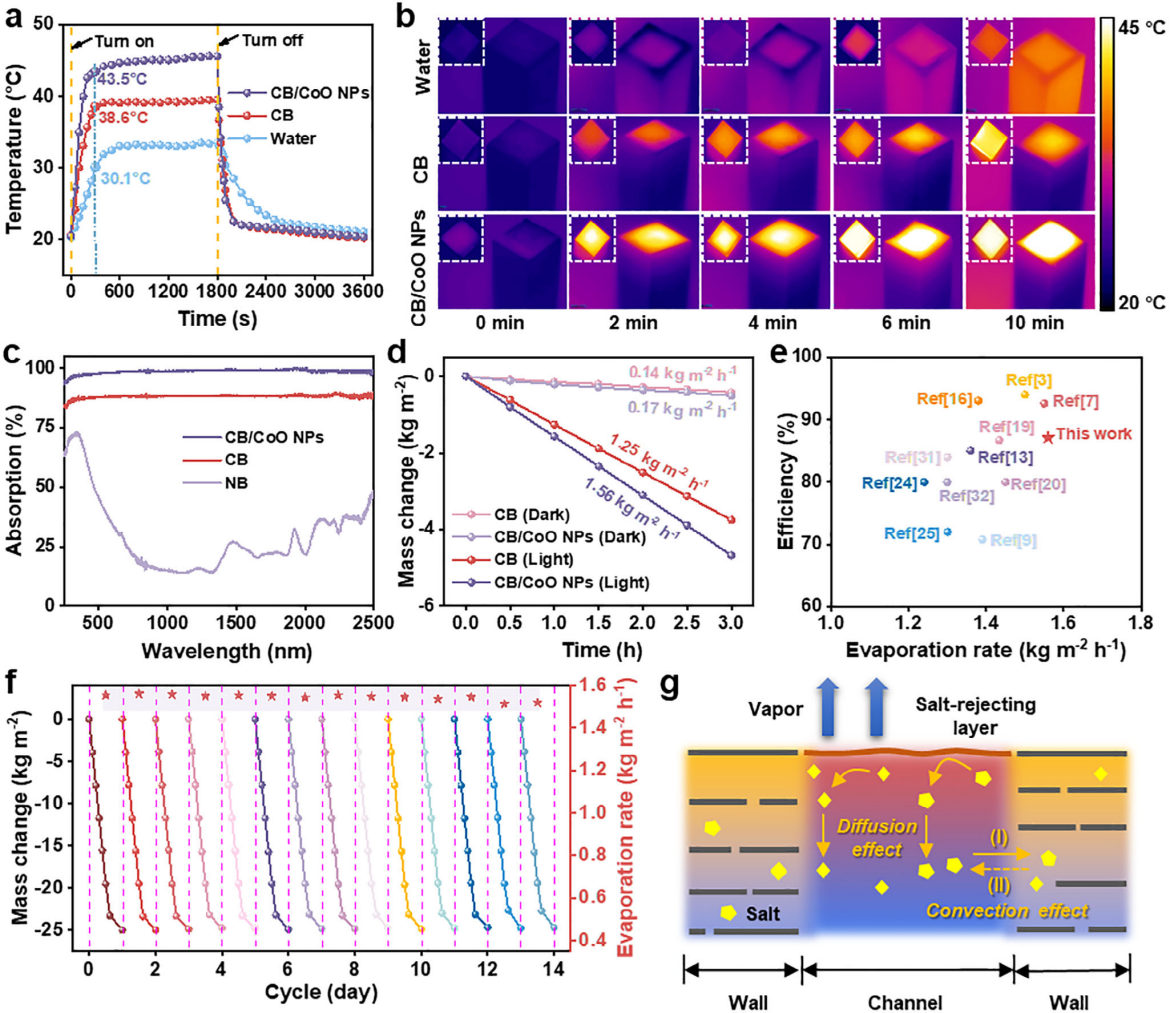
a) Time‐dependent temperature profiles of bulk water, CB, and the CB/CoO NPs‐based BSSM under 1 sun and dark conditions over 1 h. b) IR images of bulk water, CB, and the CB/CoO NPs‐based BSSM over 10 min. c) Solar absorption spectra of NB, CB, and the CB/CoO NPs‐based BSSM. d) Mass changes of the seawater via vapor evaporation under 1 sun and dark conditions over 3 h. e) Comparative water evaporation performance of the CB/CoO NPs‐based BSSM in seawater under 1 sun against the previous studies. f) Seawater mass changes and evaporation rates of the CB/CoO NPs‐based BSSM over 14 operational cycles (9 h under 1 sun and 15 h under dark conditions per cycle). g) Salt‐rejecting mechanism of the CB/CoO NPs‐based BSSM.

The related tests were conducted indoors using a calibrated solar simulator under a standard one‐sun illumination (*i.e*., 1000 W m⁻^2^) unless specially mentioned. As illustrated in **Figure** **3**a and Table S3 (Supporting Information), during the initial illumination phase (t = 0–5 min), the BSSM demonstrated a pronounced nonlinear photothermal response, with its surface temperature rising exponentially and rapidly reaching 43.5 °C within 5 min, eventually approaching thermal equilibrium. In contrast, the surface temperature of the control CB sample increased only from 20.6 to 38.6 °C, while the temperature of the pure water sample was even more gradual, reaching just 30.1 °C after 5 min. The overall temperature distribution was recorded by an infrared thermal imaging system (Figure 3b). The CB‐based system exhibited significant thermal heterogeneity, with the evaporation interface showcasing a rapid temperature rise upon solar illumination, while the underlying and lateral water regions remained nearly isothermal. In sharp contrast, the pure water system maintained a uniform temperature profile, with minimal thermal gradients between the surface and sides. This pronounced disparity is primarily attributed to the superior optical property of the CB sample (≈88.14%, Figure 3c) and the minimized heat dissipation of the interfacial evaporator. Following surface modification with CoO NPs, the CB/CoO NPs‐based BSSM test system demonstrated an even more pronounced photothermal response,^[^ ^47^, ^48^ ^]^ which can be ascribed to the BSSM's exceptional solar absorption of 98.65% (Figure 3c).

Given the above characteristics, the CB/CoO NPs‐based BSSM achieved an exceptional evaporation rate of 1.56 kg m⁻^2^ h⁻^1^ under 1 sun illumination, representing a 24.8% enhancement over the CB system (1.25 kg m⁻^2^ h⁻^1^) (Figure 3d). Besides, the photothermal conversion efficiency (*η*) of the CB/CoO NPs‐based BSSM was calculated to be as high as 87.11%, with a 1.252‐fold improvement compared to the CB (69.56%). This performance surpasses that of most recently reported non‐BSSM material systems (Figure 3e; Table S4, Supporting Information).

In seawater desalination applications, the service stability of evaporators largely depends on their salt tolerance and salt‐resistance capabilities. BSSM maintained a stable seawater evaporation performance over a 14‐day period, with evaporation rates consistently exceeding 1.51 kg m⁻^2^ h⁻^1^, well indicating its long‐term operational reliability (Figure 3f). SEM analysis (Figure S7, Supporting Information) revealed that salt crystals gradually accumulated within parenchyma cells as evaporation time extended to 15 h. However, during this process, no significant salt deposition was observed in the critical regions, like the vessel channels, tracheids, and evaporation interface (Figure S8, Supporting Information). Notably, after the 9‐h nighttime resting period, nearly all accumulated salt crystals dissolved and spontaneously flowed back into the underlying seawater, demonstrating the system's self‐regenerating capability. We further assessed the salt resistance of the BSSM through anti‐scaling performance tests. As shown in Figure S9 (Supporting Information), salt began to precipitate on the surface of the CB reference sample after 1 hour, and its surface was almost entirely covered by salt crystals within 2 h. In sharp contrast, no salt crystals were observed on the surface of the CB/CoO NPs‐based BSSM throughout the experiment. Additionally, Figure S10 (Supporting Information) demonstrates the immersion stability test results of BSSM in 3.5 wt.% artificial seawater. As the immersion time extended, the structural integrity of the well‐designed BSSM did not undergo significant alterations. Meanwhile, no noticeable change can be detected in the immersion solution, indicating that BSSM possesses excellent structural stability. This series of experimental results collectively highlights the excellent salt tolerance and salt‐resistance capabilities of the CB/CoO NPs‐based BSSM.

Indeed, the anti‐salt capability of the CB/CoO NPs‐based BSSM is primarily attributed to its hierarchical microenvironments, which leverage authigenic multi‐field synergies under solar irradiation (Figure 3g), *i*.*e*., under illumination, seawater evaporation within the vessels and tracheids will lead to localized increases in salt concentration, creating a significant concentration gradient. This gradient drives the dynamic migration of salt through dual pathways: 1) in the vertical direction, salt can spontaneously flow back into the bulk seawater via the diffusion effect, and 2) in the horizontal direction, salt can diffuse through pits into adjacent parenchyma cells containing lower‐salinity water, forming localized salt storage (Figure 3g‐I). Simultaneously, the rise in temperature during evaporation further enhances the dynamic distribution and diffusion‐driven removal of salt. Such synergistic interplay between the optimized temperature field and flow field can well improve salt migration efficiency and effectively reduce the salt concentration at the evaporation interface, thereby inhibiting salt accumulation. Under dark conditions, the system dynamics undergo a significant transformation: evaporation rate within vessels and tracheids drops sharply, facilitating an increased rate of salt exchange within the system. At this stage, the accumulated salt in the parenchyma cells forms a reverse concentration gradient with the surrounding seawater. This gradient drives the dissolution of salt crystals in the parenchyma cells through a thermodynamically spontaneous process, allowing the dissolved salt to diffuse back into the vessels and tracheids (Figure 3g‐II). Throughout this process, the flow‐field design of the BSSM promotes continuous water circulation and optimizes salt redistribution, achieving dynamic salt balance. In sharp contrast to traditional Janus evaporators, the BSSM, by precise regulation of the concentration, temperature, and flow fields, coupled with the synergistic effects of multi‐scale mass transfer mechanisms,^[^ ^49^ ^]^ maintains a stable evaporation performance during continuous solar desalination experiments.

### 2.4 Solar‐Powered Cs^+^ Extraction

**Figure 4:**
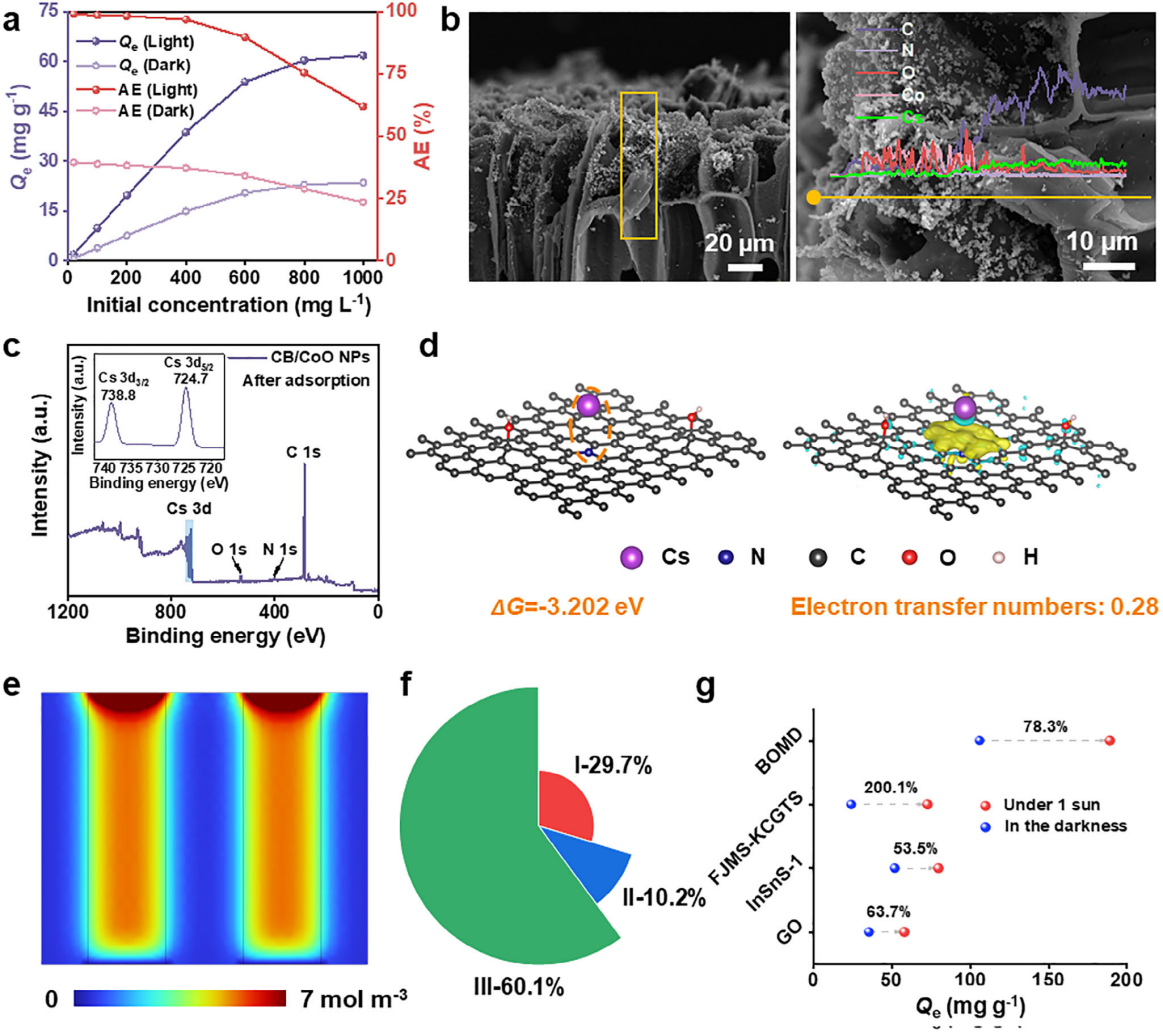
a) Effect of different initial Cs^+^ concentrations upon the CB/CoO NPs‐based BSSM's adsorption efficiency and capacity. b) SEM images and elemental line scans of the Cs^+^ adsorbed CB/CoO NPs‐based BSSM. c) XPS analysis of the Cs^+^ adsorbed CB/CoO NPs‐based BSSM. d) Scheme showcasing Cs^+^ adsorbed on CB surface and the related adsorption energy (∆G) and electron transfer number. e) COMSOL simulation of Cs^+^ distribution within the CB/CoO NPs‐based BSSM under 1 sun. f) The enhanced factor distribution of different effects: I‐Temperature field; II‐Concentration field; III‐Convection field. Initial conditions: 400 mg L^−1^ of Cs^+^ solution under 1 sun. g) The solar‐evaporation enhanced Cs^+^ extraction strategy on different reported adsorbents: GO,^[^ ^50^ ^]^ lnSnS‐1,^[^ ^51^ ^]^ FJMS‐KCGTS,^[^ ^52^ ^]^ BOMD.^[^ ^53^ ^]^ Initial conditions: 5 mg L^−1^ of Cs^+^ solution under 1 sun.

Under one‐sun illumination, the BSSM showed exceptional Cs⁺ adsorption capability (**Figure** **4**a; Table S5, Supporting Information): in low concentration range (<200 mg L⁻^1^), the adsorption efficiency approached 100%; even as the initial concentration increased to 400 mg L⁻^1^, the removal efficiency remained high at ≈96.87%, with an adsorption capacity of up to 38.75 mg g⁻^1^. The adsorption kinetics studies (Figure S11 and Table S6, Supporting Information) further clarified the nature of the adsorption process. The pseudo‐first‐order kinetic model (R^2^ = 0.99381) offers a better description of the entire adsorption phase, providing a better fit than the pseudo‐second‐order model (R^2^ = 0.98244). The ionic competitive adsorption determined the highly selective adsorption capacity of Cs^+^ (Figure S12, Supporting Information). The presence of common competing ions (*i*.*e*., Na^+^, K^+^, Mg^2+^, and Ca^2+^) in seawater has little effect on the adsorption efficiency, and BSSM still maintains a high Cs^+^ adsorption efficiency of above 80%. After Cs⁺ adsorption, the BSSM retains its original hierarchical porous structure, with CoO NPs uniformly dispersed upon the CB surface (Figure 4b), implying that the Cs⁺ adsorption process did not compromise the structural integrity of BSSM, thereby highlighting its robustness. Such excellent structural stability is primarily attributed to the strong chemical bonding between the CB matrix and CoO NPs. The line‐scan EDS analysis (Figure 4b) and XPS results (Figure 4c; Figure S13, Supporting Information) further confirm the successful adsorption of Cs⁺.

The outstanding Cs⁺ adsorption capacity of the BSSM surpassed those of many biomass‐based materials (Table S7, Supporting Information), which can be primarily attributed to two key features: 1) The abundant surface functional groups can provide ample adsorption sites. DFT simulations (Figure 4d) further provided a more in‐depth mechanistic understanding. The adsorption energy (Δ*G*) of Cs⁺ on the CB surface was calculated to be −3.202 eV, confirming that the Cs⁺ adsorption process is a thermodynamically spontaneous reaction. Moreover, an electron transfer number of 0.28 demonstrated limited electron participation during adsorption, with the primary driving forces being electrostatic interactions and coordination bonding rather than significant electron exchange. These findings collectively suggest that Cs⁺ is mainly captured within the BSSM structure via electrostatic interactions and coordination bonding. 2) Hierarchical microenvironments with authigenic multi‐field under solar irradiation. Specifically, the photothermal conversion induced by illumination can elevate the local temperature and concentration, enhancing the surface reaction kinetics (Figure 4a; Figure S14, Supporting Information). The solar‐driven evaporation process also increases the diffusion coefficient of ions, facilitating their approach to the adsorption sites and thus accelerating the adsorption reaction according to thermodynamic principles. COMSOL models (Figure S15, Supporting Information) have been established to verify this speculation. Compared to that under dark conditions (Figure S16, Supporting Information), BSSM showed a significantly increased Cs^+^ enrichment performance (Figure 4e). Under the authigenic multi‐field effects (*i.e*., temperature field, concentration field, and convection field), the Cs^+^ extraction performance of BSSM is significantly improved. The contribution values of the above three field effects to the enhancement of Cs^+^ extraction performance are 29.7%, 10.2%, and 60.1%, respectively (Figure 4f). It is worth noting that this multi‐field enhanced Cs^+^ extraction effect is also applicable to other Cs^+^ adsorbents (Figure 4g). Taking the reported BOMD as an example, 1 sun of light irradiation can increase the Cs^+^ adsorption performance by up to 78.3%, from 106 to 189 mg g⁻^1^.

### 2.5 Practical Applications

**Figure 5:**
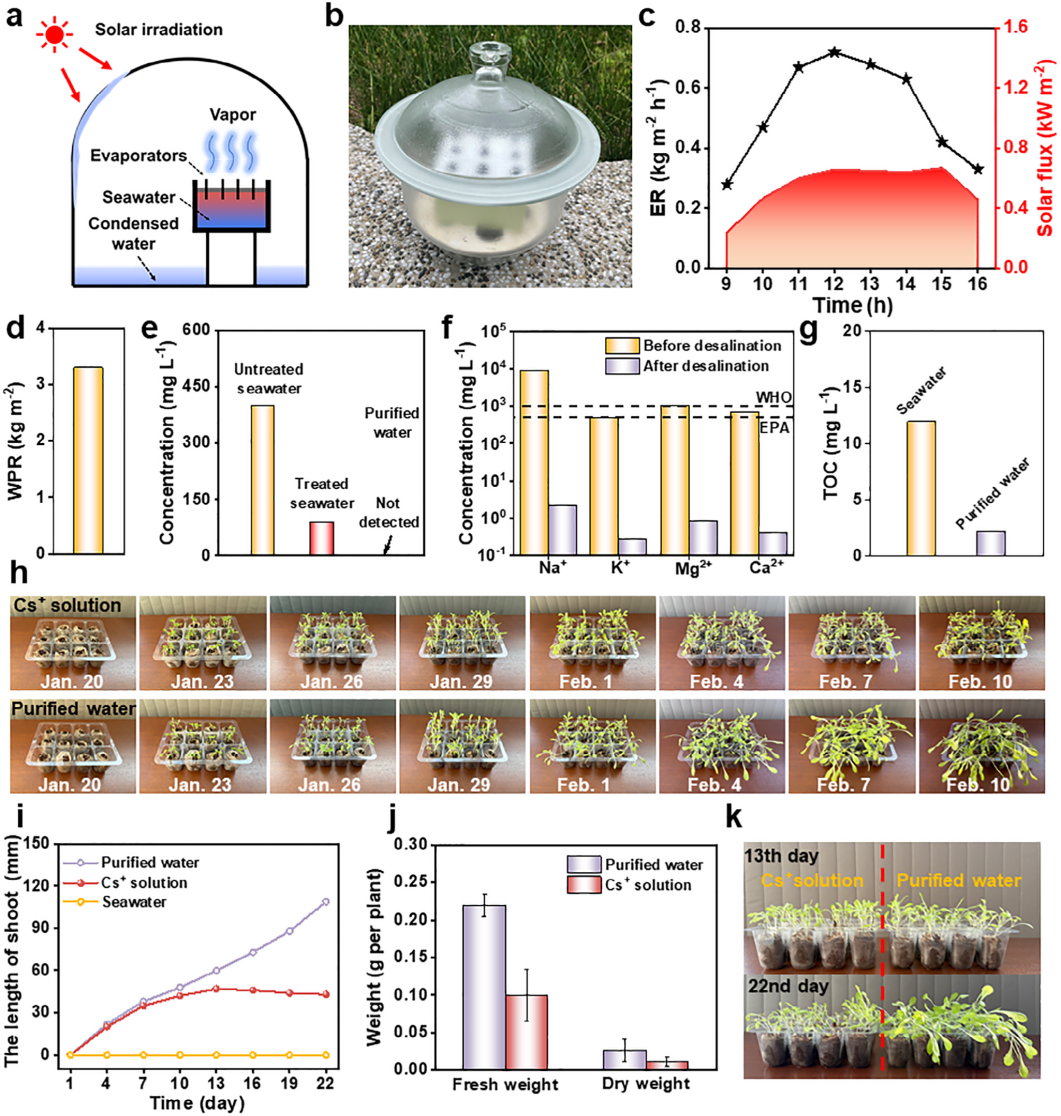
a) Schematic showing the outdoor experimental setup. b) Outdoor testing photo. c) Solar flux and evaporation rate during the outdoor test. d) Water production rate (WPR) of the CB/CoO NPs‐based BSSM. e) Cs^+^ concentrations in Bohai seawater before and after treatment, as well as in purified water. f) Ion concentrations in original seawater and purified water. g) Total organic carbon (TOC) levels in Bohai seawater and the obtained purified water. h) Digital photos of plant growth from Day 1 to Day 22, irrigated with Cs⁺ solution and purified water. i) The lengths of shoots with seawater, Cs⁺ solution, and purified water irrigation. j) Fresh weight and dry weight of the harvested plants. k) Comparison photos of lettuce growth on Day 13 and Day 22.

To further evaluate the performance of BSSM in real‐world application scenarios, we systematically investigated its water evaporation performance and Cs^+^ extraction capability under outdoor conditions. **Figure** **5**a,b exhibits the outdoor testing setup's structural design and physical photography. Figure 5c presents the outdoor test results, revealing a correlation between the evaporation rate and solar radiation intensity. Despite the relatively weak solar radiation during the testing period (Hong Kong, January 2025), the well‐designed BSSM can still operate stably, highlighting its good environmental adaptability. Over a continuous 7‐h experiment, the BSSM achieved a high clean water production rate of ≈3.3 kg m⁻^2^ (Figure 5d) and a Cs^+^ extraction capacity of ≈34.58 mg g⁻^1^ (Figure 5e). More significantly, no Cs^+^ was detected in the purified water (Figure 5e), confirming the exceptional performance of the designed BSSM in cesium resource recovery and water purification.

Moreover, to validate the seawater desalination efficacy of the designed BSSM, we further conducted a comparative quality analysis of the original seawater and the corresponding condensed water. As demonstrated in Figure 5f and Table S8 (Supporting Information), the ion concentrations in the obtained condensate were markedly reduced, all well below the maximum permissible levels for drinking water established by the World Health Organization (WHO) and the U.S. Environmental Protection Agency (EPA).^[^ ^54^, ^55^ ^]^ Furthermore, Figure 5 g and Figures S17 and S18 and Tables S9 and S10 (Supporting Information) display the levels of total organic carbon (TOC), chemical oxygen demand (COD), total dissolved solids (TDS), conductivity and pH values in domestic water, Bohai seawater, and the obtained purified water. The obtained data indicate that the BSSM is capable of effectively eliminating organic matter, reducing chemical pollutants, as well as substantially decreasing the content of dissolved solids, thus showing its remarkable aptitude for water‐quality improvement. In conclusion, these confirm the reliability of the BSSM in practical seawater desalination applications, especially for radioactive seawater. Better yet, the BSSM also demonstrates remarkable efficacy in treating organic dye‐contaminated wastewater, achieving exceptional results (Figure S19, Supporting Information).

Given the distributed clean water production characteristics of BSSM, it can be deeply integrated with urban or marine agriculture. To comprehensively assess the application potential of the BSSM in agricultural irrigation, a systematic investigation was carried out. Figure 5h details the growth of *lettuce* (*Lactuca sativa*) under two irrigation conditions: 1) simulated polluted water containing 400 mg L^−1^ Cs^+^; 2) condensed water from BSSM. In both trays, *lettuce* began to germinate on the first day (January 20, 2025), and the number gradually increased. As the growth period progressed, a comparison revealed that plants irrigated with purified water showed more lush and healthy growth, with bright‐colored leaves and relatively thick branches. Conversely, the plants irrigated with the Cs^+^‐containing solution exhibited growth retardation starting from the 10th day, with shrunken and wilted leaves and thinner branches. This outcome indicates that the condensed purified water exerts a positive influence on promoting plant growth, while the Cs^+^‐containing solution inhibits plant growth. This phenomenon can potentially be attributed to the accumulation of Cs^+^ within the plants, which subsequently inflicts physiological damage.^[^ ^56^ ^]^

To quantitatively evaluate the impacts of different water qualities on plant growth, a systematic analysis was conducted on the growth kinetic parameters of lettuce plants irrigated with seawater, Cs^+^‐containing solution, and purified water, respectively. Figure 5i depicts the dynamic trends of the height of plants irrigated with these three water sources over time. From the obtained height data (Figures S20–S23, Supporting Information), it is evident that the plants irrigated with purified water exhibited the most prominent height‐growth trend. During the 22‐day growth period, these plants grew rapidly, with an average height reaching 109 mm. In sharp contrast, the height increases of the plants irrigated with the Cs^+^ solution was relatively slow. Notably, starting from the 13th day, the plant height (with an average height of 47 mm) began to decline slightly and eventually dropped to an average height of 43 mm, further validating that Cs^+^ may disrupt plants' water absorption and growth‐regulation mechanisms. As for the plants irrigated with seawater failed to grow due to their excessively high salt concentration, and the average height remained at 0 mm. Figure 5j further compares the fresh and dry weights of the plants at the time of harvest under different water‐source irrigation conditions. Through comparative analysis, it was found that the plants irrigated with purified water significantly increased both fresh and dry weights, and their mass was more than twice that of the plants irrigated with the Cs^+^ solution. Additionally, Figure 5k visually presents the growth of lettuce irrigated with Cs^+^ solution and purified water on the 13th and 22nd days, respectively. By comparison, it can be clearly observed that the lettuce irrigated with purified water shows a significant growth advantage. Its leaves are not only brighter green and thicker, but also the overall growth form is lusher, indicating that the obtained purified water provides favorable conditions for plant photosynthesis and promotes plant metabolism and nutrient accumulation. In stark contrast, the lettuce irrigated with Cs^+^ solution showcases varying degrees of growth impediments, with leaves turning yellow and wilting, and the overall growth rate was slow, exhibiting apparent symptoms of physiological damage. The above‐mentioned experimental results effectively demonstrate the significant potential of BSSM‐purified water for application in agricultural irrigation.

### 2.6 Discussion

Some issues need further discussion:

1. Low freshwater production performance. Outdoor experiments indicate that, like most solar‐driven evaporators, the BSSM's water production rate lags behind its evaporation rate due to suboptimal condensation efficiency under ambient conditions. To address this, future iterations could integrate radiative cooling coatings or external condensation systems to enhance freshwater yield. However, in this work, we emphasize that the BSSM's unique value lies in its dual functionality: simultaneous freshwater and cesium extraction. Given the high economic value of cesium relative to pure water, evaporation‐driven Cs^+^ enrichment may outweigh the need for dedicated condensation in certain applications. From the perspective of cesium extraction, outdoor condensation devices may not be necessary because naturally forced convection outdoors could further enhance the Cs^+^ extraction rate. Indeed, the trade‐off between freshwater output and Cs^+^ extraction efficiency must be evaluated based on cost‐performance metrics and regional resource priorities.
2. Competitive adsorption and physiological toxicity. When deployed in salt lakes, the BSSM may face competitive adsorption from other interfering ions such as Ru^+^, which shares similar sorption characteristics with Cs^+^. In environments with high Ru^+^ concentrations, engineering highly selective Cs^+^‐adsorbing BSSM variants will be critical. Additionally, the ecological safety of the module requires further scrutiny. To ensure sustainability, future designs could employ biodegradable materials (*e.g*., engineered proteins) to construct the BSSM, thus minimizing environmental impact while maintaining high Cs^+^ extraction efficiency.
3. Trade‐offs in porosity optimization. The porosity of the BSSM presents a critical performance trade‐off: while higher porosity enhances water uptake, it also increases parasitic heat loss. Conversely, lower porosity restricts mass transfer, impeding both water and Cs^+^ transport. These competing effects can collectively diminish the evaporation efficiency of BSSM^[^ ^44^, ^49^ ^]^—and by extension, Cs^+^ extraction performance. Notably, the designed BSSM here leverages natural bamboo as a substrate, which inherently limits precise control over internal structural parameters (e.g., pore size distribution, tortuosity). To resolve this, future research should systematically investigate how porosity gradients, hierarchical pore architectures, or post‐synthetic modifications (e.g., controlled carbonization or chemical etching) could optimize the balance between thermal management and mass transport. Such studies would provide actionable insights for scaling BSSM variants across diverse brine sources.

## 3 Conclusion

In summary, our research comprehensively validates the dual functionality of the CB/CoO NPs‐based BSSM in the solar‐powered co‐extraction of freshwater and Cs^+^ from nuclear‐contaminated seawater. In the artificially nuclear‐contaminated seawater, the BSSM demonstrated excellent practical performance, achieving a freshwater production rate of 3.3 kg m⁻^2^ day⁻^1^ and a Cs^+^ extraction capability of 34.58 mg g⁻^1^. Going forward, incorporating an external condensation design can further improve the BSSM's outdoor performance. The proposed multi‐field synergizing theory can also be broadly applicable beyond Cs^+^ adsorbents, extending to advanced sorbents for lithium, uranium, and other critical elements by integrating various adsorption materials, thereby enabling efficient resource recovery from seawater. Moreover, when deployed for nuclear wastewater treatment, BSSM's daily processing capacity can be augmented through waste heat utilization. Eventually, the resulting purified water is suitable for direct agricultural irrigation, offering a sustainable solution to bolster food security—particularly in coastal and island nations. As such, the BSSM system developed in this work not only presents an innovative approach to addressing seawater nuclear contamination but also evidences its robust potential for application in cesium resource recovery, finally holding significant practical implications for water security and agricultural sustainability.

## Conflict of Interest

The authors declare no conflict of interest.

## Supporting information



Supporting Information

## Data Availability

The data that support the findings of this study are available from the corresponding author upon reasonable request.
