# Supplementary material for: Solar‐Powered Hierarchical Microenvironments with Authigenic Multi‐Field Synergies for Simultaneous Extraction of Freshwater and Cesium
Source: Adv Sci (Weinh). 2025 May 11;12(28):2505997. doi: 10.1002/advs.202505997 (PMC12302566; doi:10.1002/advs.202505997)
Supplement: Supplementary file 1 — Supporting Information [file ADVS-12-2505997-s001.docx]

Supporting Information

Solar-Powered Hierarchical Microenvironments with Authigenic Multi-Field Synergies for Simultaneous Extraction of Freshwater and Cesium

Liuyan Zhu,^a,#^ Lin Zhu,^b,#^ Ting Shi,^c,#^ Ke Zhao,^d^ Wenwen He,^a^ Lu Han,^a,*^ Zeying Jin,^a^ Jialin Kang,^a^ Shanfu Sun,^e^ Ningning Cao,^f,*^ and Zhen Yu^g,*^

^a^ Hebei Key Laboratory of Active Components and Functions in Natural Products, Hebei Normal University of Science & Technology, Qinhuangdao, 066004, China.

^b^ Air Defense and Antimissile School, Air Force Engineering University, Xi’an, 710100, China.

^c^ Instrumental Analysis & Research Center, Sun Yat-Sen University, Shenzhen, 518107, China.

^d^ School of Chemistry and Chemical Engineering, Beijing Institute of Technology, Beijing, 102488, China.

^e^ School of Aerospace Science and Technology, Xidian University, Xi’an, 710126, China.

^f^ College of Engineering and Applied Sciences, Nanjing University, Nanjing, 210093, China.

^g^ Department of Mechanical Engineering, City University of Hong Kong, Hong Kong, 999077, China.

^#^ These authors contributed equally to this work.

^*^ Corresponding authors: hl3395@hevttc.edu.cn, caonn1991@nju.edu.cn, zhenyu@cityu.edu.hk.

**Table of Contents**

1. Methods3

2. Supporting Figures S1~S23 9

3. Supporting Tables S1~S10 20

4. Supporting References 31

**1 Methods**

**1.1 Materials** **and reagents**

Natural bamboo (NB) was harvested from Guizhou Province, China, and air-dried naturally to a moisture content of less than 12%. Ethanol (>99.5%), cobalt (II) acetate tetrahydrate (99.5%), sodium chloride (99.5%), methyl orange (96.0%), and methylene blue (96.0%) were purchased from Sinopharm Chemical Reagent Co., Ltd., China. Considering the experimental safety and operational constraints associated with the radioactive isotope ^137^Cs, non-radioactive cesium chloride (^133^CsCl, 99.9%, Shanghai Macklin Biochemical Technology Co., Ltd.) was adopted as a cesium source substitute. All the above chemical reagents were of analytical-grade purity and applied without further purification. The ultrapure water (resistivity: 18.25 MΩ cm), prepared using a Milli-Q system, was used throughout the experiments.

**1.2 Fabrication of CB and CB/CoO NPs-based BSSM**

The natural bamboo (NB) was cut into thin slices with a thickness (*i.e*., height) of 1 or 2 cm along the direction perpendicular to its growth. The slices were ultrasonically cleaned with anhydrous ethanol and deionized water for 30 minutes each, then dried at 60 °C for 24 hours. Subsequently, the cleaned bamboo slices were carbonized in a tube furnace under an argon atmosphere at a heating rate of 10 °C min⁻^1^ from ambient to 700 °C, where they were held for 3 hours to obtain carbonized bamboo (CB).

Next, the CB was immersed in a 0.05 mol L⁻^1^ ethanol solution of cobalt (II) acetate tetrahydrate for 12 hours and then naturally dried. After that, it was heated to 500 °C in a tube furnace at a heating rate of 10 °C min⁻^1^ in an argon atmosphere. During this process, ethanol vapor was introduced at a flow rate of 20 mL min⁻^1^, and the temperature was maintained at 500 °C for 60 minutes to ensure the uniform and firm deposition of CoO NPs layer upon the surface of CB. The resulting composite material was denoted as CB/CoO NPs-based BSSM.

**1.3 Characterizations**

X-ray diffraction (XRD) analysis was performed using a D/MAX 2500 diffractometer with Cu Kα radiation to determine the crystalline phases of the materials. Fourier-transform infrared spectroscopy (FT-IR, Rigaku/H-7650) was utilized to identify the functional groups present in the materials. The morphology and microstructure of the materials were characterized using field-emission scanning electron microscopy (FE-SEM, Hitachi SU8010) and transmission electron microscopy (TEM, Hitachi H7000). The degree of graphitization was assessed using a Raman spectrometer (DXRxi) with an excitation wavelength of 532 nm. The valence states and defects of surface atoms of the obtained materials were analyzed by X-ray photoelectron spectroscopy (XPS, ThermoFisher Nexsa). The water contact angle was measured using a contact angle analyzer (Data Physics, OCA 15EC) with a high-speed camera. The ion concentrations were determined by an inductively coupled plasma optical emission spectrometer (ICP-OES, Optima 8300, PerkinElmer). The absorbance of the samples was measured using a UV-visible spectrophotometer (UV-Vis, UV-1900, Shimadzu). The optical absorption properties of the materials were evaluated using a UV-visible-near-infrared spectrophotometer (UV/Vis/NIR, UV-3600 Plus) equipped with an integrating sphere. The concentration of Cs^+^ in the filtrate was determined using an atomic absorption spectrophotometer (AAS, AA-7000, Shimadzu).

**1.4 Solar-powered water evaporation and continuous self-desalination tests**

Using the Bohai seawater in China as the working medium, the solar-powered water evaporation and continuous self-desalination capacities of the well-designed CB/CoO NPs-based BSSM were tested. A xenon lamp (CEL-HXUV300H5, China) equipped with an AM1.5 filter was used as the solar simulator. An electronic balance (FA20048, China) with a precision of 0.1 mg was employed to record the mass change of the working fluid. Besides, an infrared camera (HE640S, SEELAND) was used to monitor and record the temperature distribution during evaporation. The solar-to-vapor conversion efficiency (*η*) was calculated using the formula referenced from the literature.^[1-5]^ To systematically evaluate the practical performance of the CB/CoO NPs-based BSSM, we conducted a 14-day solar desalination cycling experiment using Bohai seawater. Each cycle simulated the typical summer sunlight conditions in Qinhuangdao, consisting of a 15-hour continuous solar evaporation phase (04:30 to 19:30) followed by a 9-hour dark resting phase. Fresh seawater was used for each cycle to ensure experimental consistency.

**1.5 Cs^+^ adsorption experiment**

Before the experiment, a 1000 mg L⁻^1^ CsCl stock solution was prepared, and Cs^+^ solutions with the desired concentrations were obtained *via* appropriate dilution. After the adsorption time had elapsed, the solution was filtered through a 0.45-μm membrane using a syringe, and the filtrate was saved for subsequent analysis. Each experiment was repeated three times to ensure reproducibility, and the average value was taken as the basis for data analysis.

The Cs adsorption capacity (*Q_t_*) at the time (*t*) is calculated using the following formula:

| $\text{Q}\text{t}\text{ =}\frac{\text{(}\text{C}\text{0}\text{–}\text{C}\text{t}\text{)×}\text{V}}{\text{m}}$ | (1) |
| --- | --- |

where *C*_0_ (mg L^‒1^) is the initial concentration of Cs^+^, C*_t_* (mg L^‒1^) is the concentration of Cs^+^ in the solution at the adsorption time of *t*, *V* (mL) is the volume of the Cs^+^ solution, and *m* (g) is the mass of the adsorbent used in the experiment.

The adsorption performance is represented by the following Cs^+^ adsorption efficiency (AE%):

| $\text{AE\% =}\frac{\text{(}\text{C}\text{0}\text{–}\text{C}\text{t}\text{)}}{\text{C}\text{0}}\text{×100\%}$ | (2) |
| --- | --- |

To investigate the adsorption kinetics mechanism of CB/CoO NPs-based BSSM, pseudo-first-order and pseudo-second-order kinetic models were employed, which are as follows:

The adsorption rate constant can be calculated by the pseudo-first-order kinetic model:

| $\text{log (}\text{Q}\text{e}\text{–}\text{Q}\text{t}\text{) =log }\text{Q}\text{e}\text{–}\frac{\text{k}\text{1}\text{ }\text{t}}{\text{2.303}}$ | (3) |
| --- | --- |

where *Q*_e_ and *Q_t_* (mg g^–1^) are the adsorption amounts of Cs^+^ at equilibrium and at the time of *t*, respectively, and *k*_1_ is the adsorption rate constant (min^–1^). The pseudo-first-order rate constant *k*_1_ and the theoretical *Q*_e_ value can be calculated from the slope and intercept of the plot of log (*Q*_e_–*Q_t_*) against *t*.

The pseudo-second-order kinetic model can be expressed by the following equation:

| $\frac{\text{t}}{\text{Q}\text{t}}\text{=}\frac{\text{1}}{\text{k}\text{2}\text{Q}\text{e}\text{2}}\text{+}\frac{\text{1}}{\text{Q}\text{e}}\text{t}$ | (4) |
| --- | --- |

where the theoretical equilibrium adsorption capacity *Q*_e_ (mg g^–1^) and the pseudo-second-order rate constant *k*_2_ (g mg^–1^ min^–1^) can be calculated from the slope and intercept of the plot of *t*/*Q_t_* against *t*, respectively.

The contribution of the different factors to enhanced Cs^+^ adsorption under solar irradiation was calculated as follows:

| $\text{ED}_{\text{i}}\text{=}\frac{\text{q}_{\text{i}}\text{-}\text{q}_{\text{d}}}{\text{q}_{\text{s}}\text{-}\text{q}_{\text{d}}}$ | (5) |
| --- | --- |

where $\text{ED}_{\text{i}}$ was contribution of the different enhanced factors; $\text{q}_{\text{i}}$was the adsorption capacity of BSSM in the dark under different conditions (like different concentration and different temperature); $\text{q}_{\text{s}}$ was the adsorption capacity under solar irradiation; $\text{q}_{\text{d}}$ was the adsorption capacity in the dark at 25 °C.

**1.6 Outdoor experiment**

The outdoor experiments were conducted on the City University of Hong Kong campus during winter to assess the system’s performance under seasonal conditions. Initially, seawater samples were collected from the Bohai Sea, and the Cs^+^ concentration was adjusted to 400 mg L^–1^ to prepare the experimental solution. Subsequently, artificial seawater was prepared in a measured amount, and the CB/CoO NPs-based BSSM was deployed for water evaporation performance and Cs^+^ extraction testing under solar irradiation. Throughout the experiment, solar radiation intensity was continuously monitored, and variations in evaporation rates were systematically recorded to ensure the accuracy and reproducibility of the results. During a 7-hour continuous evaporation trial, the mass of generated freshwater was collected and measured, while the Cs^+^ extraction capacity was determined and quantified to evaluate system performance.

**1.7 Plant irrigation experiment**

The plant irrigation experiment was carried out on the laboratory balcony of Hebei Normal University of Science and Technology. A seedling tray has twelve separate compartments for plant growth. Lettuce seeds (*i*.*e*., high-quality lettuce, Shouhe) were selected for demonstration, and standard seedling blocks (*i.e*., the substrate nutrient soil, Shouhe) were applied for plant cultivation. Every day at 8:00 PM, 5 ml of the corresponding water was supplied to each compartment. At 2:00 PM, the growth of the plants was recorded with a camera. The plants were harvested after 20 days of growth. After washing the soil attached to the roots, the wet biomass of the plants was recorded. The dry biomass was determined after dehydration at 80 °C for 12 h.

**1.8 DFT simulation**

Density functional theory (DFT) calculations were performed using the Vienna Ab-Initio Simulation Package (VASP), leveraging its integrated graphical interface for setup, execution, and analysis.^[6]^ Core electrons were described through the projected augmented wave (PAW) method, and the exchange-correlation energy was treated within the framework of the generalized gradient approximation (GGA) using the Perdew-Burke-Ernzerhof (PBE) functional.^[7]^ A plane wave basis set with a cutoff energy of 500 eV was employed, while a 3×3×5 Monkhorst-Pack k-point grid was used for structural optimizations and electronic structure calculations. The conjugate gradient method was employed to fully optimize the positions of the system until all forces on each atom were minimized to below 0.02 eV/Å. The energy convergence of the whole self-consistent process was determined based on 10^–5^ eV. The Gaussian smearing with a width of 0.05 eV was applied to ensure proper electronic state broadening.^[8]^

**1.9 COMSOL simulation**

A transient model was employed to study the Cs^+^ adsorption process under solar irradiation and dark conditions. The geometric model is shown in Fig. S15. The details can be found in our early reported works.^[9, 10]^

**2 Supporting Figures S1~S23**


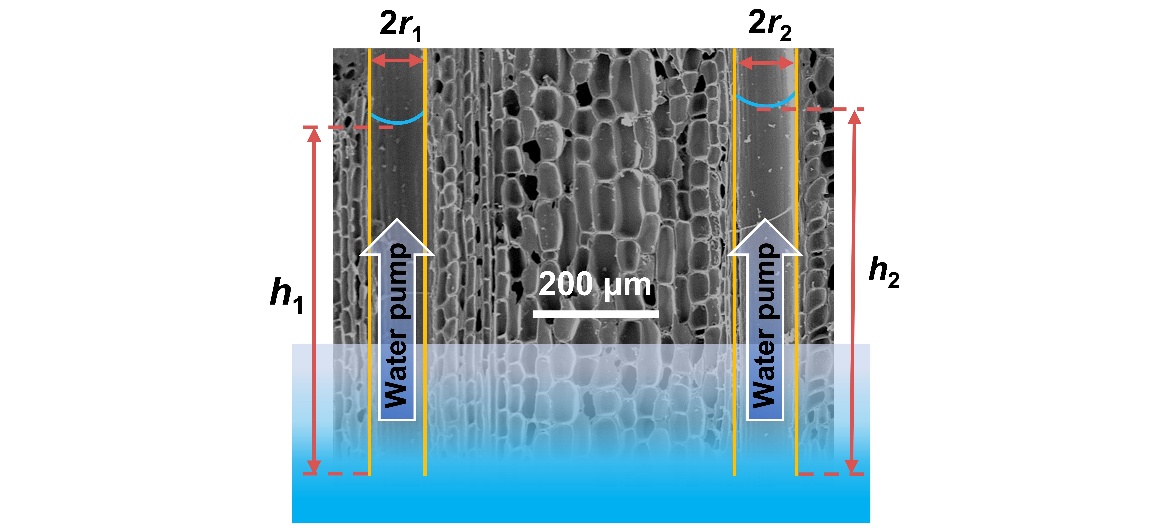


**Figure S1.** Schematic showing the water equilibrium heights in CB vessel channels with different radiuses.


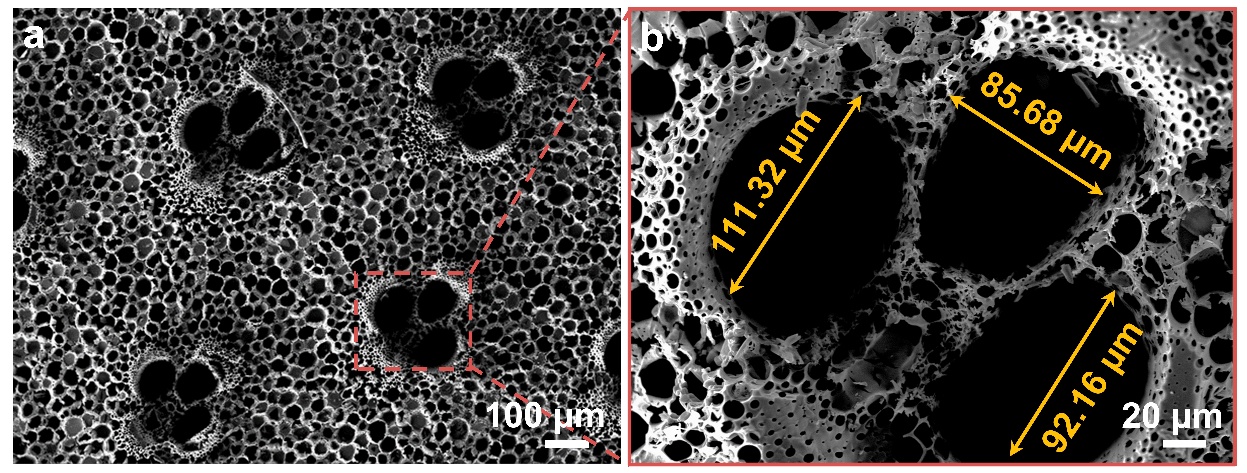


**Figure S2.** SEM images of the vessel channels within the CB.


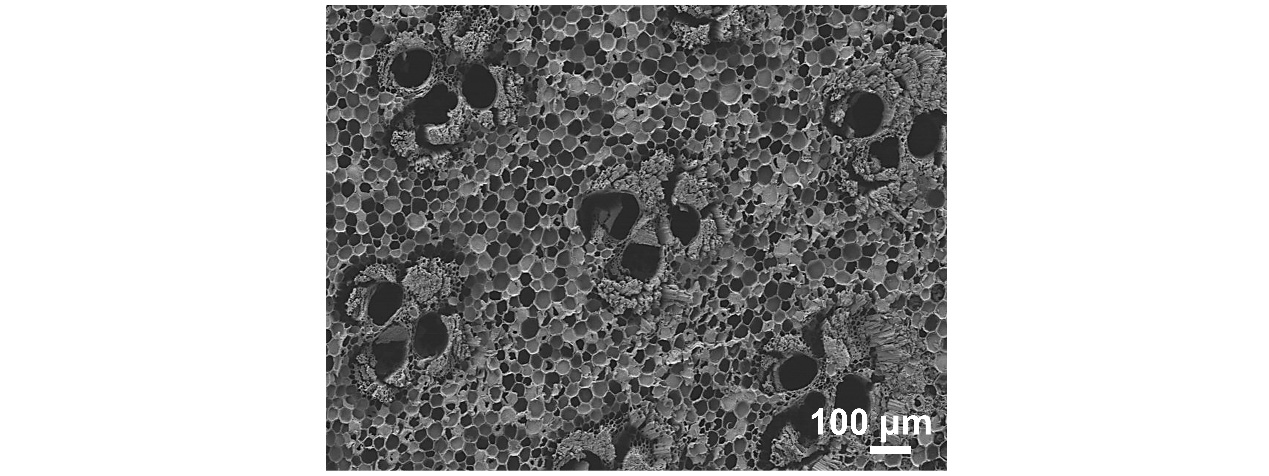


**Figure S3.** SEM image of the cross-section of the original NB.


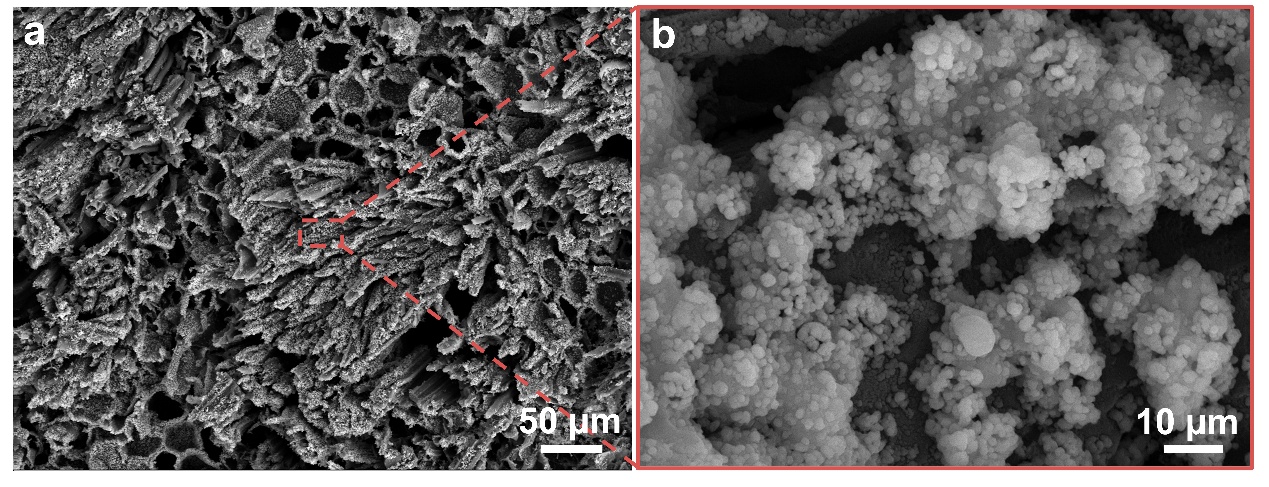


**Figure S4.** SEM images of the cross-section of the CB/CoO NPs-based BSSM.


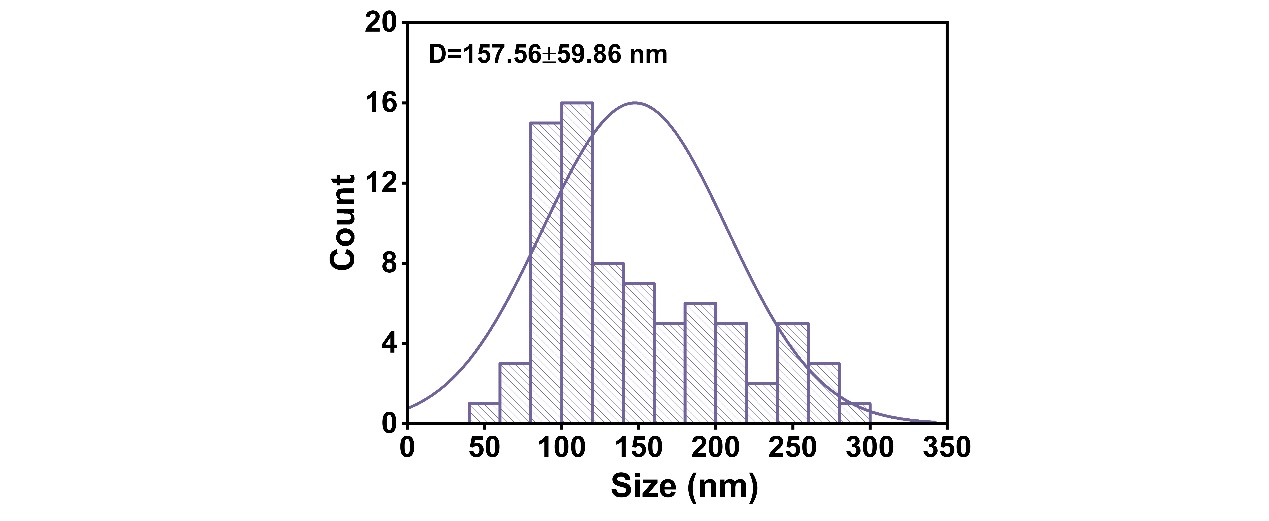


**Figure S5.** Size distribution of the CoO NPs upon the CB/CoO NPs-based BSSM.


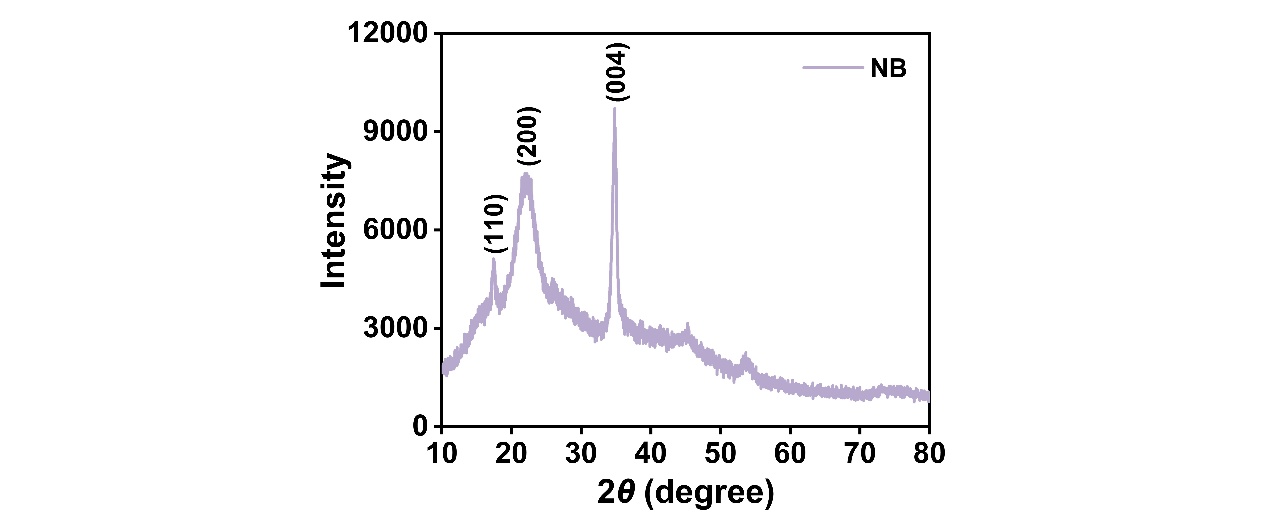


**Figure S6.** XRD pattern of the NB.


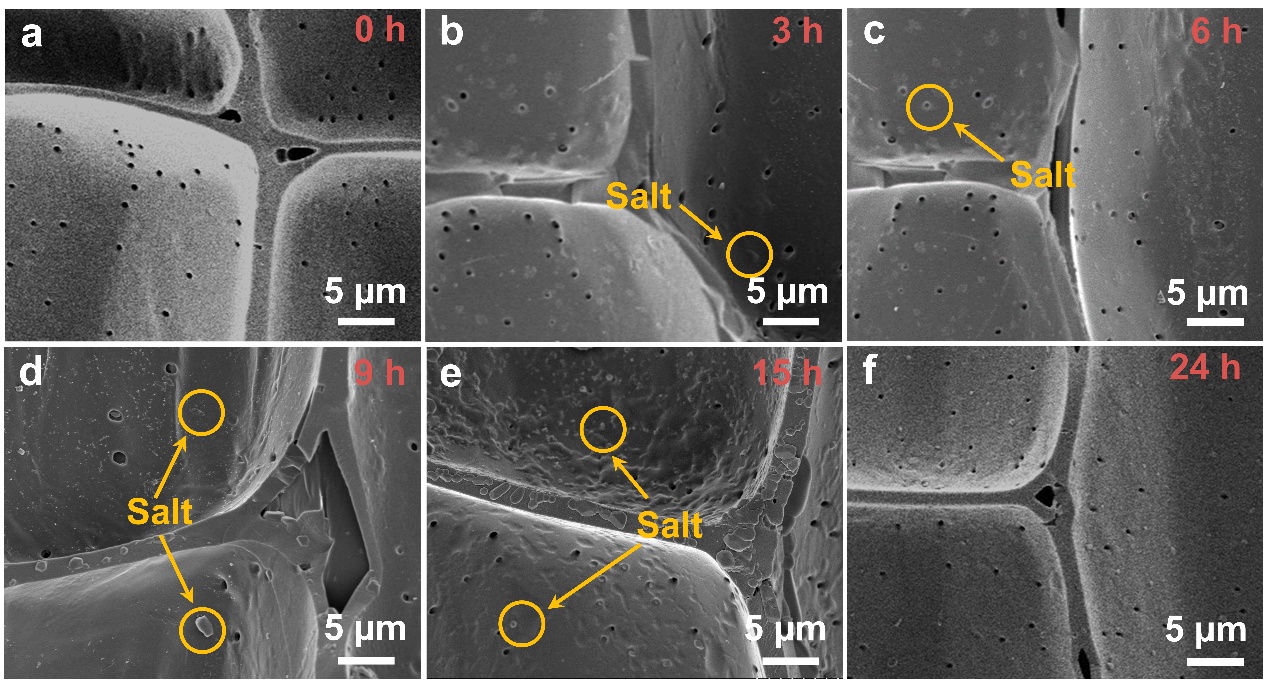


**Figure S7.** SEM images of salt crystallization in the parenchyma cell within the CB/CoO NPs-based BSSM during the 14th cycle.


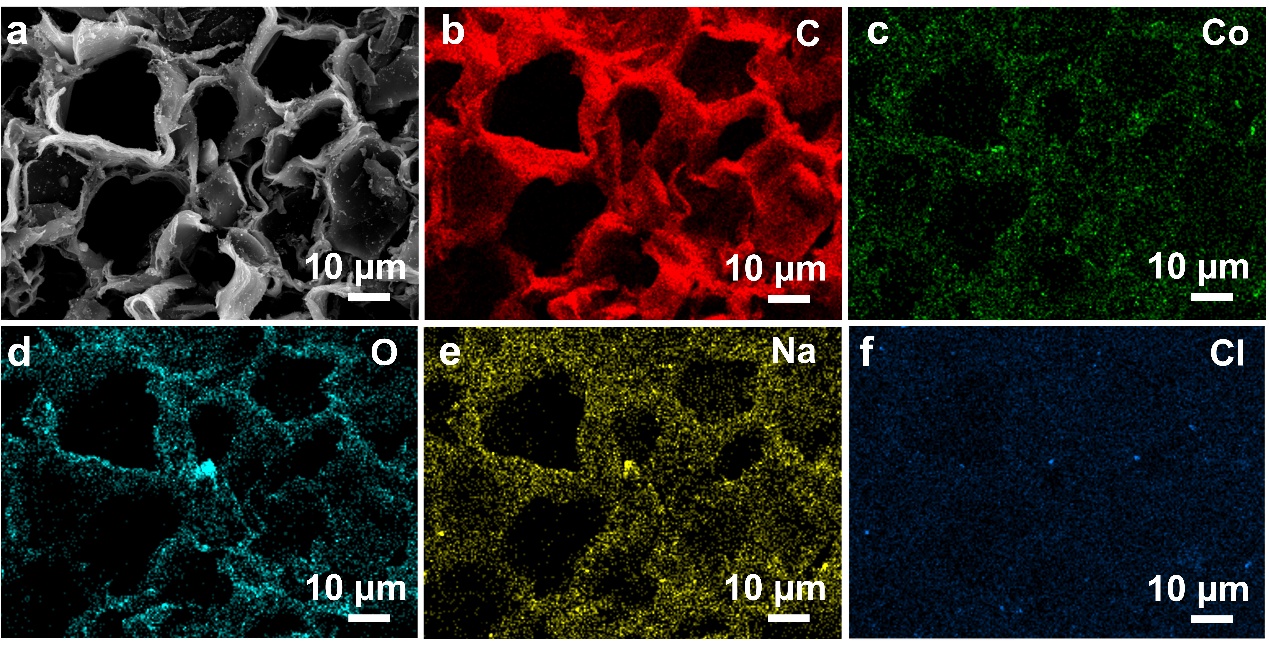


**Figure S8.** SEM image and the corresponding elemental mappings of the CB/CoO NPs-based BSSM’s surface after ongoing desalination for the 14th cycle.


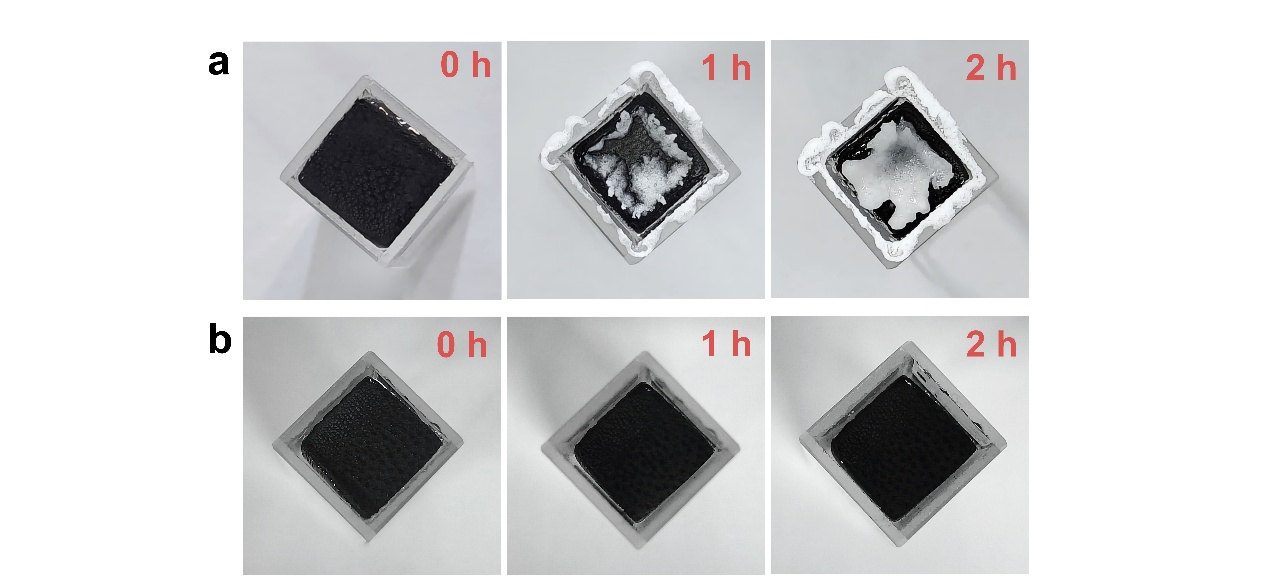


**Figure S9.** Salt crystallization behavior of a) the pristine CB and b) the CB/CoO NPs-based BSSM when treating the 20 wt% NaCl solution.


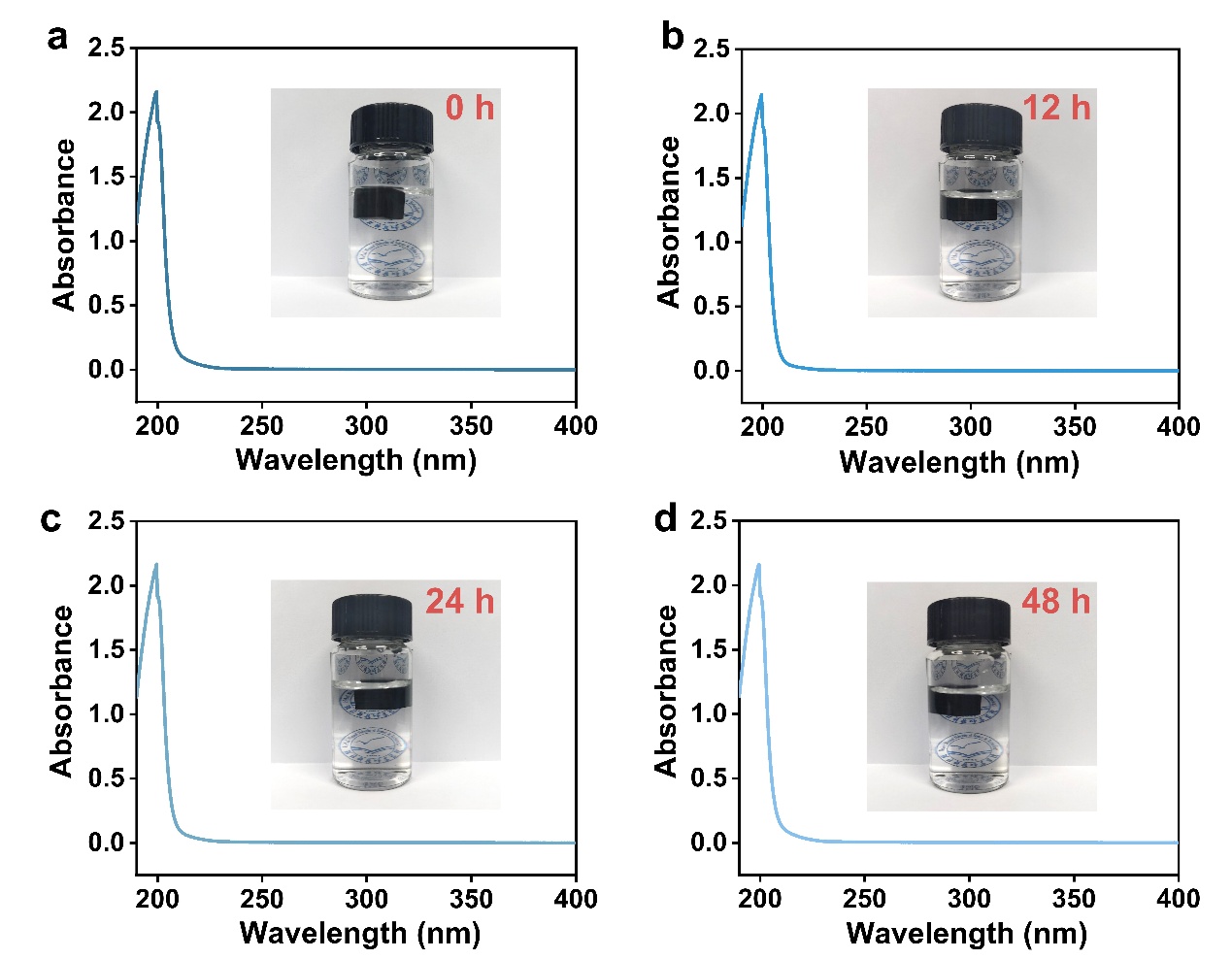


**Figure S10.** UV-Vis absorption spectra of the corresponding impregnation solution of the CB/CoO NPs-based BSSM after soaking in 3.5 wt% NaCl artificial seawater for 0 h, 12 h, 24 h, and 48 h (insets showing the corresponding digital photos).


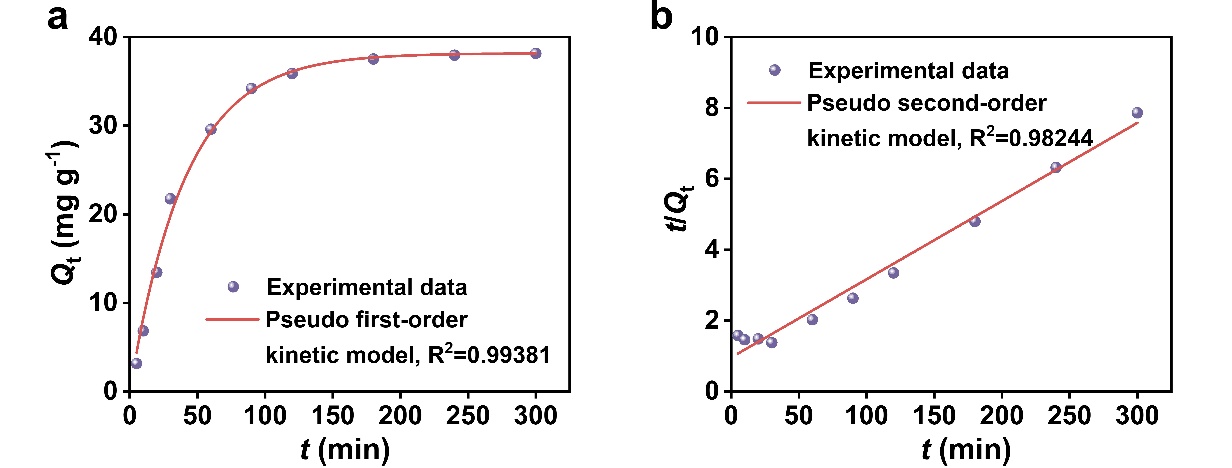


**Figure S11.** a) Pseudo-first-order, and b) pseudo-second-order kinetic models for Cs^+^ adsorption by the CB/CoO NPs-based BSSM.


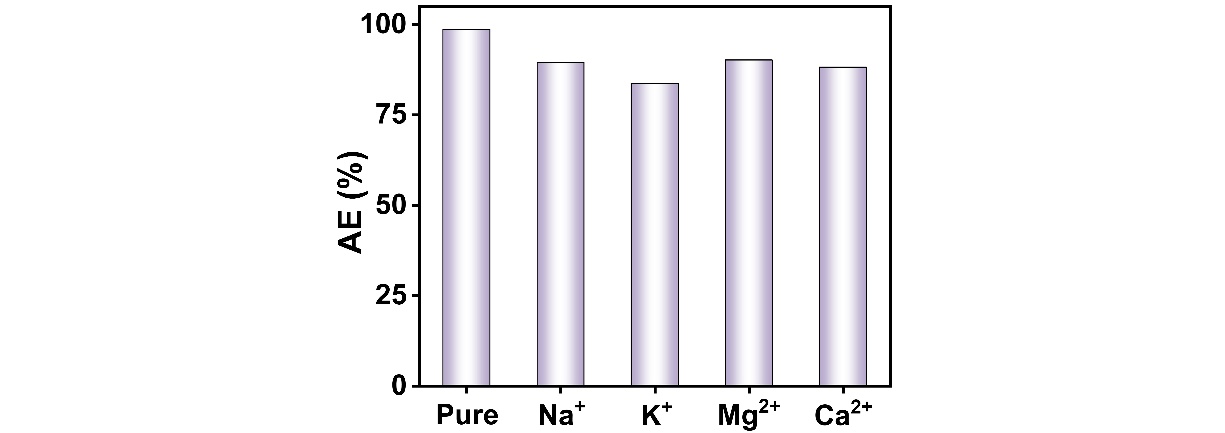


**Figure S12.** Effect of the competing ions (*i.e*., Na⁺, K⁺, Mg²⁺, Ca²⁺) on the Cs^+^ adsorption efficiency by the CB/CoO NPs-based BSSM.


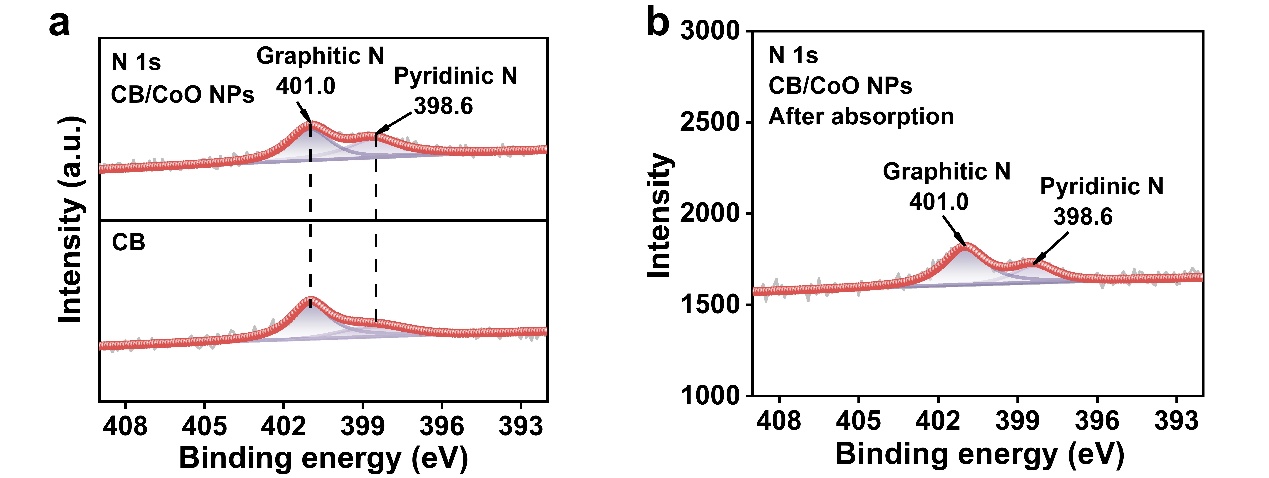


**Figure S13.** a) N 1s XPS spectra and peak fitting of the pristine CB and the CB/CoO NPs-based BSSM. b) N 1s XPS spectra and peak fitting after Cs^+^ adsorption by the CB/CoO NPs-based BSSM.


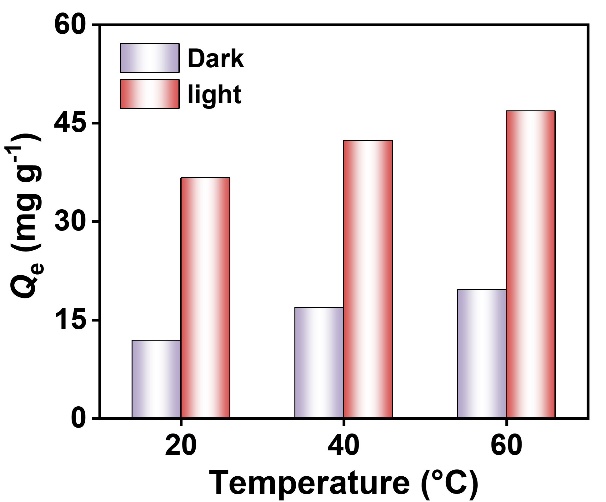


**Figure S14.** Effect of different temperatures on the adsorption capacity of Cs^+^ by the CB/CoO NPs-based BSSM.


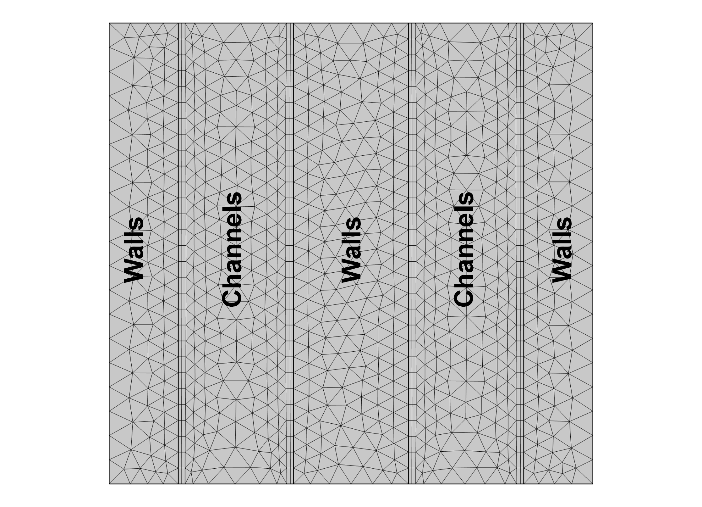


**Figure S15.** The geometric model of COMSOL simulation.


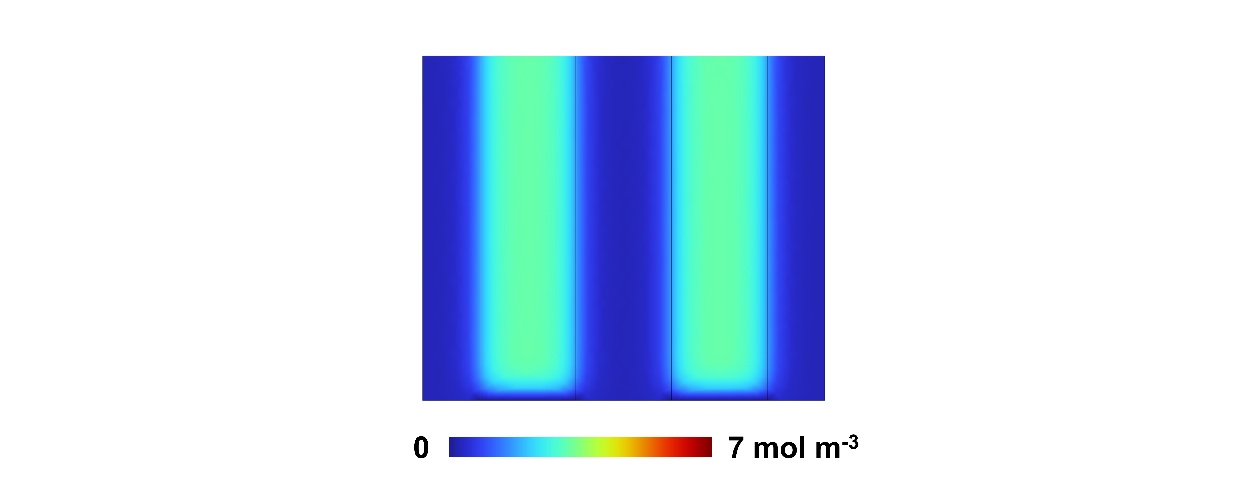


**Figure S16.** The COMSOL simulation of Cs^+^ distribution within the CB/CoO NPs-based BSSM under dark conditions.


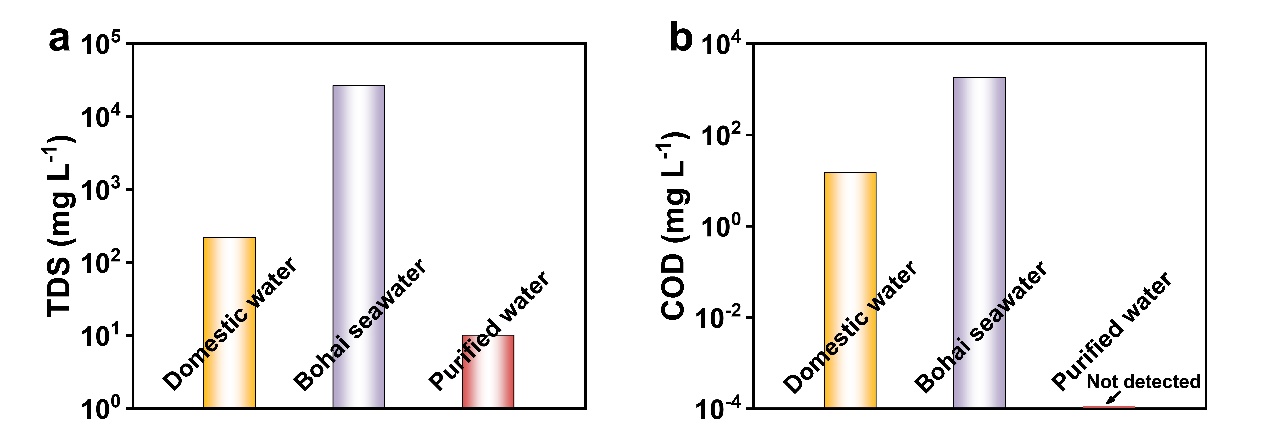


**Figure S17.** a) TDS and b) COD values in the domestic water, Bohai seawater, and purified water.


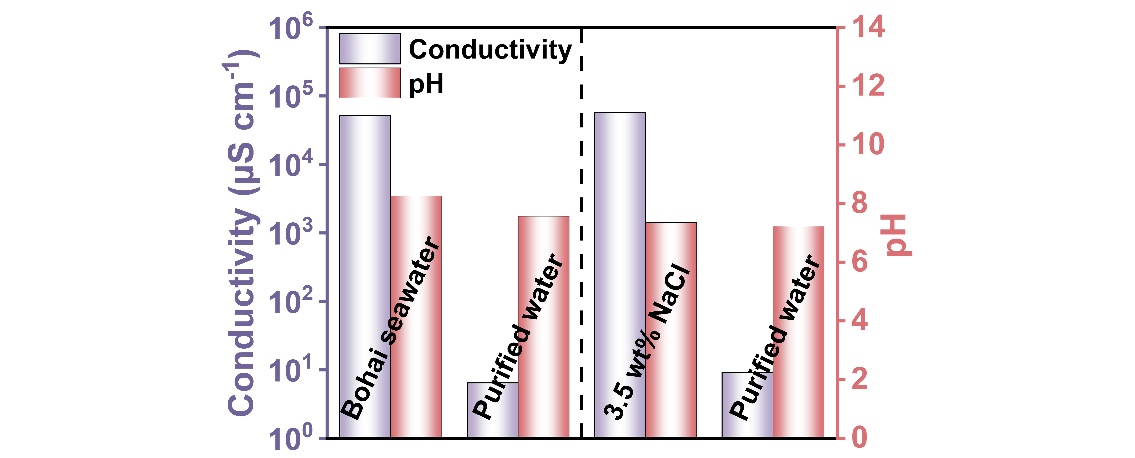


**Figure S18.** Changes in conductivity and pH of the Bohai seawater and 3.5 wt% NaCl artificial seawater before and after the CB/CoO NPs-based BSSM treatment.


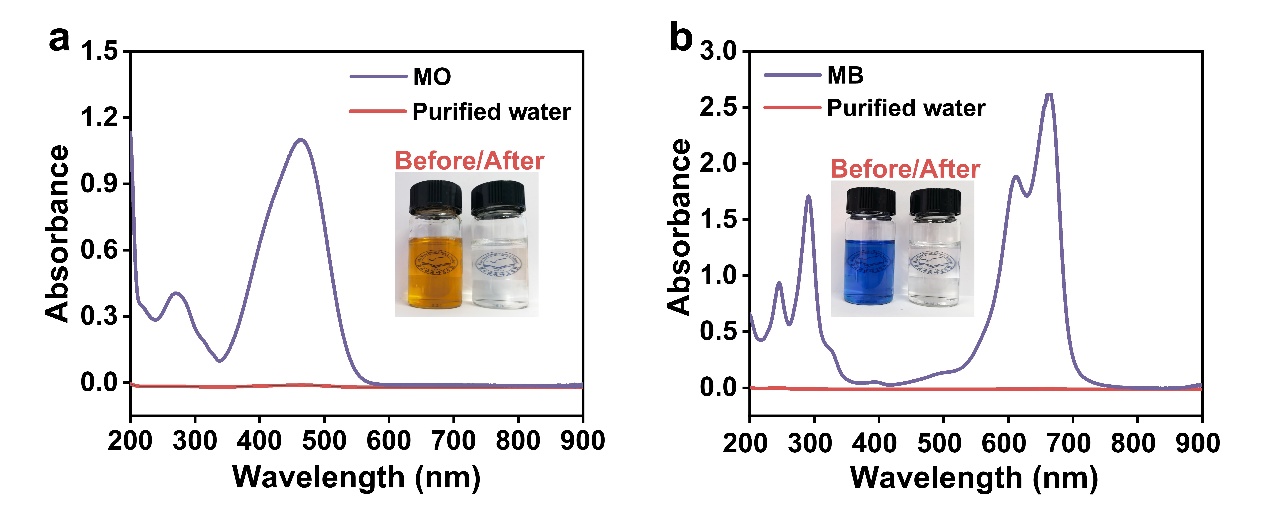


**Figure S19.** UV-Vis absorption spectra of a) MO solution and b) MB solution before and after purification (insets showing the corresponding digital photos).


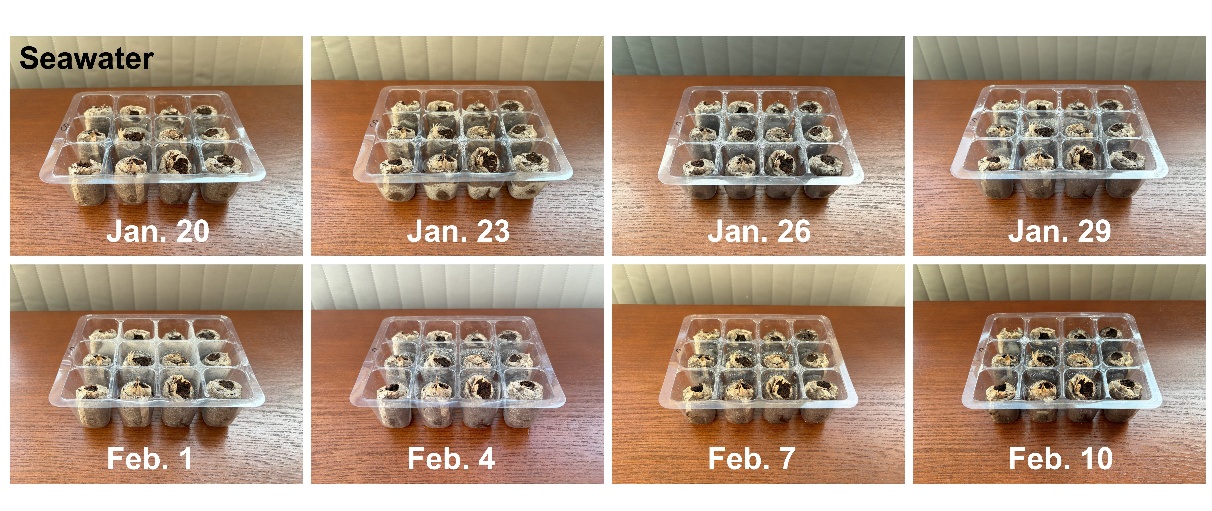


**Figure S20.** Digital photos of plant growth from Day 1 to Day 22, irrigated with the Bohai seawater.


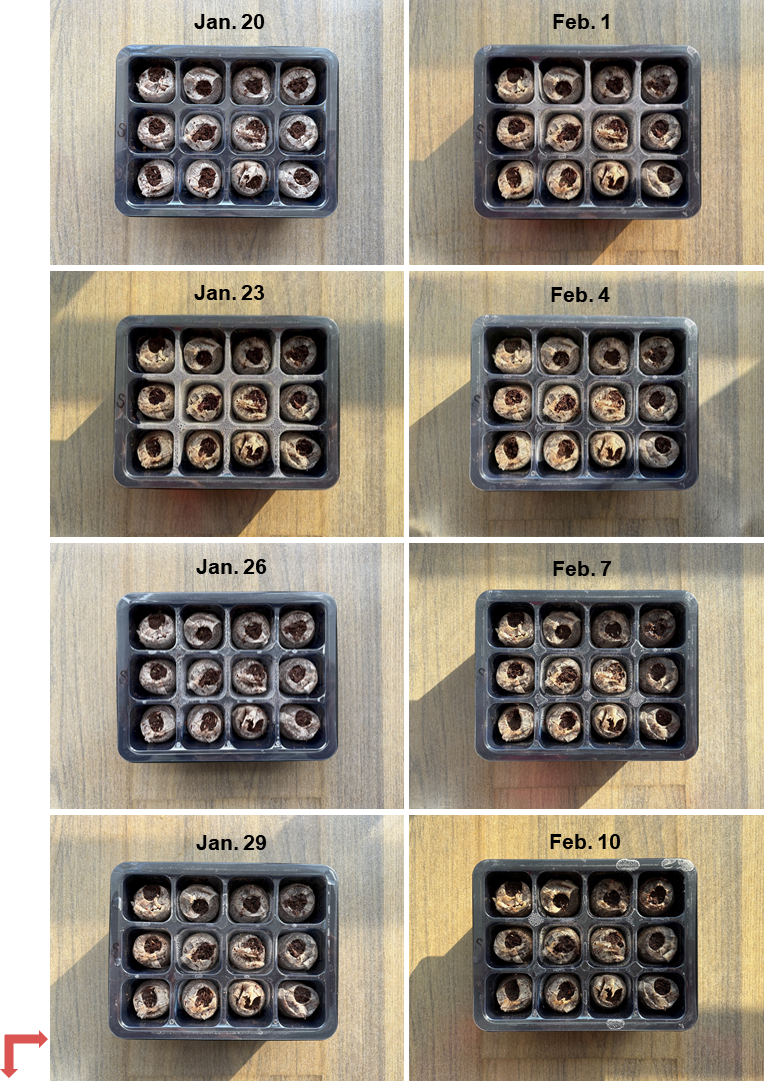


**Figure S21.** Overhead photos showcasing the growth of plants from Day 1 to Day 22, irrigated with the Bohai seawater.


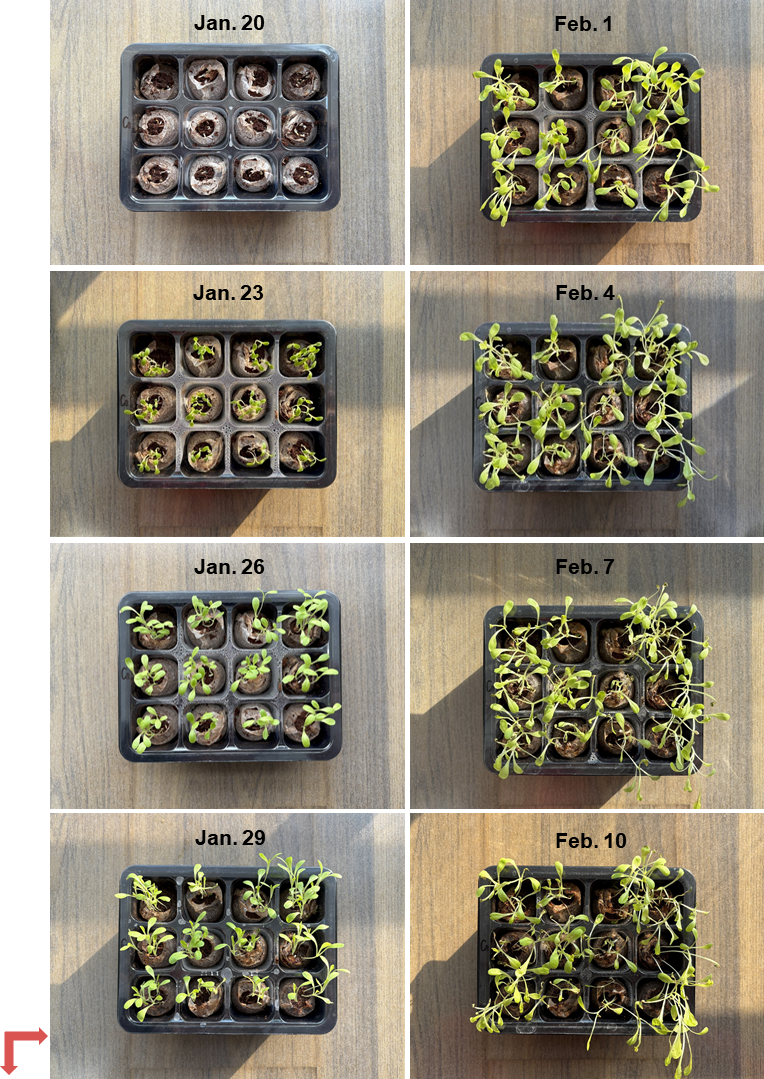


**Figure S22.** Overhead photos showcasing the growth of plants from Day 1 to Day 22, irrigated with the Cs⁺ solution.


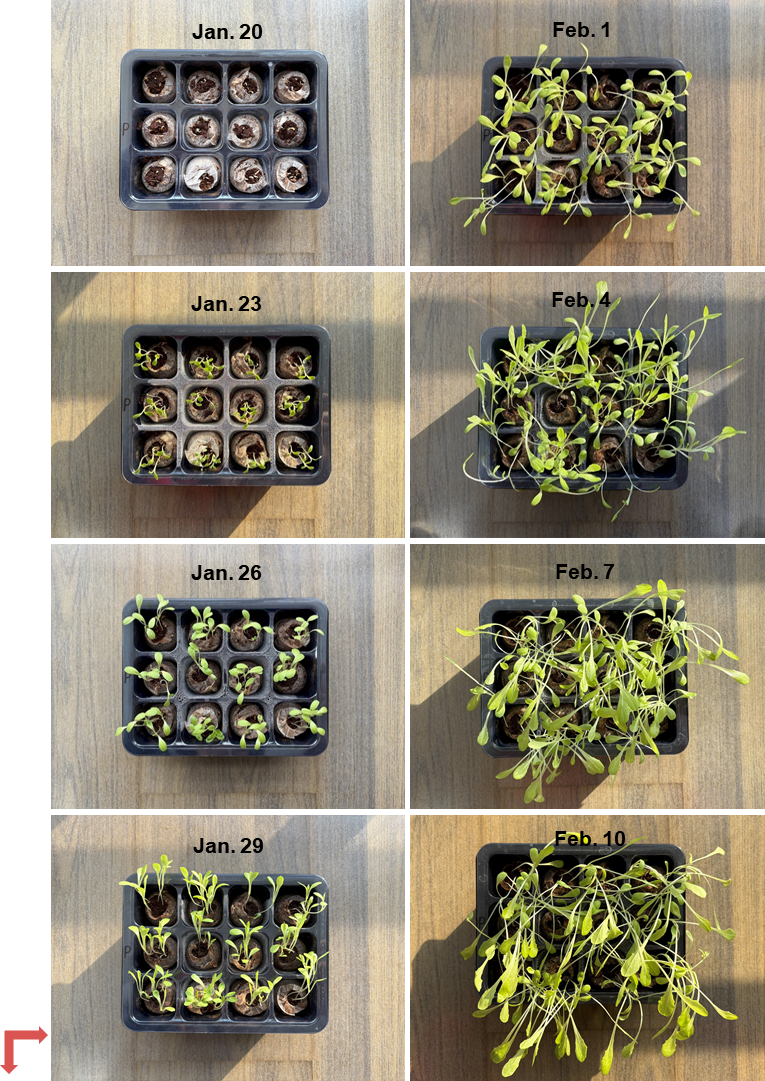


**Figure S23.** Overhead photos showcasing the growth of plants from Day 1 to Day 22, irrigated with purified water.

**3 Supporting Tables S1~S10**

| **Category** | **Method** | **Key differences** | **Advantages** | **Limitations** |
| --- | --- | --- | --- | --- |
| Seawater desalination  (Can not extract cesium) | Distillation  ^[11, 12]^ | Evaporation and condensation process | 1 Suitable for high-salinity seawater;  2 Produces high-quality water. | 1 High energy consumption and significant equipment investment;  2 High operating cost. |
|  | Reverse osmosis  ^[13, 14]^ | Semipermeable membrane to separate water and salts under high pressure | 1 Low operation cost and energy consumption;  2 Suitable for large-scale production. | 1 Strict pretreatment;  2 Membranes fouling;  3 Salinity limit. |
|  | Electrodialysis  ^[15, 16]^ | Ion-exchange membranes to separate water and salts under an electric field | 1 Moderate energy consumption;  2 Suitable for low and medium-salinity water. | 1 Less efficient for high-salinity seawater;  2 Complex equipment and high maintenance cost. |
| Cesium extraction  (Can not produce clean water) | Chemical precipitation  ^[17, 18]^ | Uses chemical reagents to form insoluble cesium precipitates for removal. | 1 Simple operation;  2 relatively low cost. | 1 Low selectivity;  2 Co-precipitate with other ions;  3 Secondary pollution. |
|  | Ion exchange  ^[19, 20]^ | Uses specific ion exchange materials to selectively adsorb cesium ions. | 1 High selectivity;  2 high recovery efficiency. | 1 Expensive ion exchangers;  2 Requires regeneration treatment;  3 Generate waste liquid. |
|  | Solvent extraction^[21, 22]^ | Uses organic solvents to selectively extract cesium from seawater. | 1 High separation efficiency;  2 Handle large water volumes. | 1 Potentially toxic solvent;  2 Complex operation;  3 Secondary pollution. |
| **This work**  **(Can extract water and cesium simultaneously)** | **BSSM** | **Integrate the interfacial evaporation and adsorption process.** | **1 Low operation cost and energy consumption;**  **2 High operation efficiency;**  **3 Suitable for various scenes;**  **4 Environmentally friendly.** | **Still in the research phase; industrial application requires further validation.** |

**Table S1.** Comparison of different methods for seawater desalination and cesium extraction.

**Table S2.** Theoretical equilibrium heights of vessel channels with varying radii within the CB.

| *r* (µm) | *θ* (°) | *h* (cm) |
| --- | --- | --- |
| 42.84 | 27.10 | 308.75 |
| 46.08 |  | 287.01 |
| 55.66 |  | 237.60 |
| **48.19 (average)** |  | **277.79 (average)** |

**Table S3.** Surface temperatures of the bulk water, the pristine CB, and the CB/CoO NPs-based BSSM under solar irradiation.

| Time (s) | Water | | CB | | CB/CoO NPs | |  |
| --- | --- | --- | --- | --- | --- | --- | --- |
| 0 | | 20.3 | | 20.6 | | 20.5 | |
| 50 | | 21.7 | | 24.2 | | 27.3 | |
| 100 | | 23.1 | | 30.1 | | 35.0 | |
| 150 | | 24.6 | | 33.1 | | 40.1 | |
| 200 | | 26.3 | | 35.7 | | 42.6 | |
| 250 | | 28.1 | | 37.5 | | 43.1 | |
| 300 | | 30.1 | | 38.6 | | 43.5 | |
| 400 | | 31.9 | | 39.1 | | 44.2 | |
| 500 | | 32.5 | | 39.2 | | 44.4 | |
| 600 | | 33.1 | | 39.1 | | 44.7 | |
| 700 | | 33.1 | | 39.2 | | 44.8 | |
| 800 | | 33.3 | | 39.2 | | 44.9 | |
| 900 | | 33.1 | | 39.1 | | 45.0 | |
| 1000 | | 33.2 | | 39.2 | | 45.1 | |
| 1100 | | 33.2 | | 39.1 | | 45.1 | |
| 1200 | | 33.1 | | 39.3 | | 45.1 | |
| 1300 | | 33.0 | | 39.2 | | 45.2 | |
| 1400 | | 33.1 | | 39.3 | | 45.3 | |
| 1500 | | 33.2 | | 39.3 | | 45.5 | |
| 1600 | | 33.5 | | 39.4 | | 45.5 | |
| 1700 | | 33.4 | | 39.5 | | 45.6 | |
| 1800 | | 33.5 | | 39.5 | | 45.6 | |
| 1820 | | 33.1 | | 36.7 | | 38.5 | |
| 1840 | | 32.6 | | 33.5 | | 34.2 | |
| 1860 | | 32.1 | | 31.1 | | 30.5 | |
| 1880 | | 31.5 | | 29.0 | | 28.2 | |
| 1900 | | 30.4 | | 26.9 | | 25.5 | |
| 2000 | | 28.5 | | 22.6 | | 22.5 | |
| 2100 | | 26.5 | | 22.0 | | 22.0 | |
| 2200 | | 25.2 | | 21.8 | | 21.9 | |
| 2300 | | 24.1 | | 21.6 | | 21.8 | |
| 2400 | | 23.4 | | 21.5 | | 21.7 | |
| 2500 | | 22.8 | | 21.4 | | 21.6 | |
| 2600 | | 22.4 | | 21.3 | | 21.5 | |
| 2700 | | 22.2 | | 21.2 | | 21.4 | |
| 2800 | | 22.1 | | 21.0 | | 21.3 | |
| 2900 | | 21.9 | | 20.9 | | 21.2 | |
| 3000 | | 21.8 | | 20.8 | | 21.0 | |
| 3100 | | 21.6 | | 20.7 | | 20.9 | |
| 3200 | | 21.5 | | 20.6 | | 20.8 | |
| 3300 | | 21.4 | | 20.5 | | 20.7 | |
| 3400 | | 21.3 | | 20.3 | | 20.6 | |
| 3500 | | 21.2 | | 20.3 | | 20.5 | |
| 3600 | | 21.1 | | 20.2 | | 20.4 | |

**Table S4.** Comparison of solar-powered water evaporation performance for different evaporators.

| Evaporator | Evaporation rate  (kg m^–2^ h^–1^) | Temperature  (°C) | Efficiency  (%) | Ref. |  |
| --- | --- | --- | --- | --- | --- |
| CNTs@SiO_2_ | 1.50 | 34.0 | 94.00 | [23] |  |
| Ag/PPy | 1.55 | 42.8 | 92.60 | [24] |  |
| PDMS-CNPs | 1.39 | 66.0 | 70.80 | [25] |  |
| PPP-MGA | 1.38 | 43.0 | 93.04 | [26] |  |
| CNF/CNT | | 1.24 | 43.5 | 80.00 | [27] |
| CB/PMMA | | 1.30 | 60.0 | 72.00 | [28] |
| C- potato | 1.36 | 39.7 | 85.00 | [29] |  |
| C-corncobs | 1.43 | 40.0 | 86.70 | [30] |  |
| Biochar-based | 1.30 | 44.5 | 84.00 | [31] |  |
| Luffa sponge | 1.30 | 36.2 | 79.98 | [32] |  |
| PS/rGO ﬁlm | 1.45 | 30.7 | 80.00 | [2] |  |
| **CB/CoO NPs** | **1.56** | **45.6** | **87.11** | **This work** |  |

**Table S5.** Effect of the initial concentrations on Cs^+^ adsorption by the CB/CoO NPs-based BSSM.

| Initial concentration  (mg L^–1^) | *Q*_e_ (Light)  (mg g^–1^) | *Q*_e_ (Dark)  (mg g^–1^) | AE (Light)  (%) | AE (Dark)  (%) |
| --- | --- | --- | --- | --- |
| 20 | 1.98 | 0.79 | 99.01 | 39.55 |
| 100 | 9.85 | 3.89 | 98.54 | 38.92 |
| 200 | 19.65 | 7.66 | 98.25 | 38.31 |
| 400 | 38.75 | 14.92 | 96.86 | 37.31 |
| 600 | 53.85 | 20.46 | 89.75 | 34.10 |
| 800 | 60.35 | 23.01 | 75.44 | 28.77 |
| 1000 | 61.85 | 23.56 | 61.85 | 23.56 |

**Table S6.** Sorption rate constants for the pseudo-first-order and pseudo-second-order kinetic models.

| Pseudo-first-order kinetic model | | |  | Pseudo-second-order kinetic model | | | |
| --- | --- | --- | --- | --- | --- | --- | --- |
| *Q*_e_^exp a^ (mg g^–1^) | 38.16 |  | | | *Q*_e_^exp a^ (mg g^–1^) | 38.16 |  |
| *k*_1_ (min^–1^) | 0.0244 |  | | | *k*_2_ (g mg^-1^ min^–1^) | 0.000506 |  |
| *Q*_e_^cal b^ (mg g^–1^) | 38.17128 |  | | | *Q*_e_^cal b^ (mg g^–1^) | 45.33092 |  |
| R^2^ | 0.99381 |  | | | R^2^ | 0.98244 |  |

^a^ denotes the equilibrium sorption capacity as estimated from experimental data.

^b^ denotes the equilibrium sorption capacity calculated from the kinetic model.

**Table S7.** Comparison of the maximum Cs^+^ adsorption by our designed CB/CoO NPs-based BSSM and the other bio-adsorbents in the literature.

| Bio-adsorbent | Concentration  (mg L^–1^) | *Q*_max_  (mg g^–1^) | Ref. |
| --- | --- | --- | --- |
| Functional modified walnut shell | 100 | 6.00 | [33] |
| Modified walnut shell | 300 | 4.94 | [34] |
| Nickel hexacyanoferrate modified coir pith | 200 | 54.32 | [35] |
| Waste biomass | 132.91 | 10.11 | [36] |
| NaOH treated moss | 1600 | 17.00 | [37] |
| Ti_3_C_2_T_x_@biochar-PDA/PEI | / | 40.30 | [38] |
| Modified pine cone | 250 | 8.74 | [39] |
| Nitric acid-modiﬁed bamboo charcoal | 800 | 45.87 | [40] |
| **CB/CoO NPs (Dark)** | **1000** | **23.56** | **This work** |
| **CB/CoO NPs (Light)** | **1000** | **61.85** | **This work** |

**Table S8.** The concentration of four major cations in Bohai seawater before and after treatment.

| Primary cations | Before (mg L^–1^) | After (mg L^–1^) |
| --- | --- | --- |
| Na^+^ | 8785.87 | 2.15 |
| K^+^ | 487.56 | 0.28 |
| Mg^2+^ | 1035.78 | 0.87 |
| Ca^2+^ | 690.56 | 0.42 |

**Table S9.** TOC, TDS, and COD values in domestic water, Bohai seawater, and purified water.

| Sample | TOC (mg L^–1^) | TDS (mg L^–1^) | COD (mg L^–1^) |
| --- | --- | --- | --- |
| Domestic water | 15.17 | 218.36 | 15. 02 |
| Bohai seawater | 11.98 | 26380.12 | 1800.45 |
| **Purified water** | **2.27** | **10.01** | **Not detected** |

**Table S10.** Conductivity and pH values of water (*i.e*., Bohai seawater and 3.5 wt% NaCl artificial seawater) treated by the CB/CoO NPs-based BSSM.

| Sample | Conductivity (μS cm^–1^) | pH |
| --- | --- | --- |
| Bohai seawater | 51300.12 | 8.25 |
| Purified water | 6.35 | 7.56 |
| 3.5 wt% NaCl | 56913.07 | 7.35 |
| Purified water | 8.95 | 7.22 |

**4 Supporting References**

[1] N. Cao, S. Lu, R. Yao, C. Liu, Q. Xiong, W. Qin, X. Wu, *Chem. Eng. J.* **2020**, 397, 125522.

[2] X. Li, W. Xu, M. Tang, L. Zhou, B. Zhu, S. Zhu, J. Zhu, *Proc. Natl. Acad. Sci.* **2016**, 113, 13953-13958.

[3] Y. Kuang, C. Chen, S. He, E.M. Hitz, Y. Wang, W. Gan, R. Mi, L. Hu, *Adv. Mater.* **2019**, 31, 1900498.

[4] N. Cao, S. Lu, Y. Yao, C. Liu, P. Zhang, Q. Xiong, Y. Li, X. Wu, *Chem. Eng. J.* **2022**, 430, 133043.

[5] H. Ghasemi, G. Ni, A.M. Marconnet, J. Loomis, S. Yerci, N. Miljkovic, G. Chen, *Nat. Commun.* **2014**, 5, 4449.

[6] G. Kresse, J. Furthmüller, *Comput. Mater. Sci.* **1996**, 6, 15-50.

[7] J. P. Perdew, K. Burke, M. Ernzerhof, *Phys. Rev. Lett.* **1996**, 77, 3865.

[8] K. Zhao, L. X. Zhang, X. Heng, L. F. Liu, B. Tang, L. J. Bie, *Nanoscale* **2022**, 14, 10980-10991.

[9] S. Xu, K. Zhao, Y. Zhou, K. Zheng, Z. Wang, Z. Yu, N. Cao, X. Liu, *Nano Energy* **2024**, 131, 110232.

[10] Y. Su, R. Gu, Y. Li, W. Wu, Z. Yu, S. Cheng, *Adv. Funct. Mater.* **2025**, 2420651.

[11] G. Tiwari, H. Singh, R. Tripathi, *Solar Energy* **2003**, 75, 367-373.

[12] A. Deshmukh, C. Boo, V. Karanikola, S. Lin, A.P. Straub, T. Tong, D.M. Warsinger, M. Elimelech, *Energy Environ. Sci.* **2018**, 11, 1177-1196.

[13] I.G. Wenten, *Desalination* **2016**, 391, 112-125.

[14] Y.J. Lim, K. Goh, M. Kurihara, R. Wang, *J. Membr. Sci.* **2021**, 629, 119292.

[15] A. Galama, M. Saakes, H. Bruning, H. Rijnaarts, J. Post, *Desalination* **2014**, 342, 61-69.

[16] G. Doornbusch, M. Tedesco, J. Post, Z. Borneman, K. Nijmeijer, *Desalination* **2019**, 464, 105-114.

[17] M.A. Soliman, G.M. Rashad, M.R. Mahmoud, *Chem. Eng. J.* **2015**, 275, 342-350.

[18] H. Rogers, J. Bowers, D. Gates Anderson, *J. Hazard. Mater.* **2012**, 243, 124-129.

[19] M.A. Lilga, R.J. Orth, J.P. Sukamto, S.D. Rassat, J.D. Genders, R. Gopal, *Sep.* *Purif. Technol.* **2001**, 24, 451-466.

[20] B. Pangeni, H. Paudyal, K. Inoue, K. Ohto, H. Kawakita, S. Alam, *Chem. Eng. J.* **2014**, 242, 109-116.

[21] P. Crowther, F. Moore, *Anal. Chem.* **1963**, 35, 2081-2085.

[22] W.J. McDowell, G.N. Case, J.A. McDonough, R.A. Bartsch, *Anal. Chem.* **1992**, 64, 3013-3017.

[23] X. Dong, L. Cao, Y. Si, B. Ding, H. Deng, *Adv. Mater.* **2020**, 32, 1908269.

[24] Y. Xu, J. Ma, Y. Han, H. Xu, Y. Wang, D. Qi, W. Wang, *Chem. Eng. J.* **2020**, 384, 123379.

[25] X. Li, S. Tanyan, S. Xie, R. Chen, Q. Liao, X. Zhu, X. He, *Sep. Purif. Technol.* **2022**, 292, 120985.

[26] Z. Chen, Y. Luo, Q. Li, X. Chen, *ACS Appl.* *Mater. Interfaces* **2021**, 13, 40531-40542.

[27] R. Hu, J. Zhang, Y. Kuang, K. Wang, X. Cai, Z. Fang, W. Huang, G. Chen, Z. Wang, *J. Mater. Chem. A* **2019**, 7, 15333-15340.

[28] W. Xu, X. Hu, S. Zhuang, Y. Wang, X. Li, L. Zhou, S. Zhu, J. Zhu, *Adv. Energy Mater.* **2018**, 8, 1702884.

[29] Y. Chen, L. Cheng, Q. Liu, M. Chen, C. Li, L. Wang, J. Shen, P. Senin, S. Yan, T. Bian, *Appl. Surf. Sci.* **2024**, 656, 159667.

[30] T. Chen, H. Xie, X. Qiao, S. Hao, Z. Wu, D. Sun, Z. Liu, F. Cao, B. Wu, X. Fang, *ACS Appl. Mater. Interfaces* **2020**, 12, 50397-50405.

[31] L. Yang, G. Chen, N. Zhang, Y. Xu, X. Xu, *ACS Sustainable Chem. Eng.* **2019**, 7, 19311-19320.

[32] A.M. Saleque, S. Ma, S. Ahmed, M.I. Hossain, W. Qarony, Y.H. Tsang, *Adv. Sustainable Syst.* **2021**, 5, 2000291.

[33] D. Ding, Z. Lei, Y. Yang, C. Feng, Z. Zhang, *J. Hazard. Mater.* **2014**, 270, 187-195.

[34] D. Ding, Y. Zhao, S. Yang, W. Shi, Z. Zhang, Z. Lei, Y. Yang, *Water Res.* **2013**, 47, 2563-2571.

[35] H. Parab, M. Sudersanan, *Water Res.* **2010**, 44, 854-860.

[36] C. Chen, J. Wang, *J. Hazard. Mater.* **2008**, 151, 65-70.

[37] M.V. Balarama Krishna, S.V. Rao, *Sep. Purif. Technol.* **2004**, 38, 149-161.

[38] F. Liu, S. Wang, C. Zhao, B. Hu, *Biochar* **2023**, 5, 2524-7867.

[39] A.E. Ofomaja, A. Pholosi, E.B. Naidoo, *Ecol. Eng.* **2015**, 82, 258-266.

[40] S. Khandaker, T. Kuba, S. Kamida, *J. Environ. Chem. Eng.* **2017**, 5, 1456-1464.
